# Supplementary material for: The link between lymphocyte subpopulations in peripheral blood and metabolic variables in patients with severe obesity
Source: PeerJ. 2023 Jun 13;11:e15465. doi: 10.7717/peerj.15465 (PMC10274585; doi:10.7717/peerj.15465)
Supplement: Supplemental Information 2 [file peerj-11-15465-s002.pdf]

STATISTICAL ANALYSIS METHOD REPORT

The link between lymphocyte subpopulations in peripheral blood and metabolic variables in patients with severe obesity

To Peer Journal #2023:01:81490:0:3:REVIEW

1- We analyzed the results after divided the participants into classes of obesity based on TBF percentages.

2- We evaluated 124 individuals with a mean age of 34.3 ± 8.8 years (Table 1S), most of whom were women (64.5%) (Table 2S). Overall, 21.0% (n = 26) of the participants formed the control group (normal TBF percentage), 20.2% (n = 25) had class 1 and 2 obesity, 25.0% (n = 31) had class 3 obesity, and 33.8% (n = 42) had class 4 obesity (Table 3S). There were no significant differences in age and sex between the groups (Table 4S).

Table 1S. **Descriptive Statistics**

	n	Minimum	Maximum	Mean	Deviation
edad (year)	124	18	56	34.31	8.810

Table 2S. **Descriptive Statistics**

sex	n	male	female
#	124	80	44
%	100	64.5	35.5

Table 3S. **Descriptive Statistics**

Entire study group		Cohorts by TBF			
		Normal body fat	Class 1 and 2 obesity	Class 3 obesity	Class 4 obesity
n	124	26	25	31	42
%	100	21.0	20.2	25	33.8

Table 4S. **two-way ANOVA**[a]

Model		Sum of squares	gl	root mean square	F	Significance
1	Regression	118.949	2	34.310	1.559	.073[b]
	Residue	1785.046	8	9.038		
	Total	1903.995	9			

a. Dependent variable: Age

b. predictors: (Constant), sex 0=male, 1=female

3- We used the adjusted Kolmogorov–Smirnov method to determine the normality of the data. We used the Phyton Environment v3.6.7 statistical package (CreateSpace 2009, USA) (Table 5S supplementary)

Table 5S. **Variables passed the normality test using adjusted Kolmogorov–Smirnov method.**

Variable	Lilliefors significance correction	
Age	0.059961084	Acepted
Weight (kg)	0.064643741	Acepted
WC (cm)	0.235672402	Acepted
TBF (%)	0.054471089	Acepted
FFM (%)	0.054471089	Acepted
Total cholesterol (mg/dL)	0.718629794	Acepted
LDL-c (mg/dL)	0.603645429	Acepted
Monocytes (%)	0.059000999	Acepted
Granulocytes (%)	0.055301976	Acepted
Total lymphocytes (%)	0.147601999	Acepted

CD4+ T lymphocytes (%)	0.164300012	Acepted
CD4+CD62+ T lymphocytes (%)	0.317973484	Acepted
CD4+CD62- T lymphocytes (%)	0.098739778	Acepted
CD8+CD28+ T lymphocytes (%)	0.22980821	Acepted
CD8+CD28- T lymphocytes (%)	0.146408573	Acepted
CD8+CD45RA+ T lymphocytes (%)	0.524792443	Acepted
CD8+CD45RA+CD45RO+ T lymphocytes (%)	0.098234112	Acepted
Abbreviations: WC, Waist circumference; TBF, Total body fat; FFM, fat free mass; LDL-c, low-density cholesterol; CD3+, T cells; TCD4+, helper T cells; TCD4+CD62+, non-effector helper T cells; TCD4+CD62-, effector helper T cells; TCD8+CD28+, no activated cytotoxic T cells; TCD8+CD28-, activated cytotoxic T cells; CD8+CD45RA+CD45RO+ in transition from naive to memory; TCD8+CD45RO+, memory cytotoxic T cell.		

4- We transformed the variables that did not pass the normality test to achieve a normal distribution. We used the Phyton Environment v3.6.7 statistical package (CreateSpace 2009, USA) (Table 6S supplementary).

Table 6S. **Methods used to transform variables and normalize their distribution.**

Variable	Transformation method
BMI (kg/m2)	ln(x)
VF (cm2)	ln(x)
FFM (kg)	1/sqrt(x)
Glucose (mg/dL)	1/(x^2)
HbA1c (mg/dL)	1/(x^2)
Insulin (µUI/mL)	sqrt(x)
HDL-c (mg/dL)	1/sqrt(x)
Triglycerides (mg/dL)	1/sqrt(x)
CRP (mg/dL)	sqrt(x)
SBP (mmHg)	ln(x)
DBP (mmHg)	sqrt(x)
Leukocytes (cel/µL)	ln(x)
Monocytes (cel/µL)	1/sqrt(x)
Granulocytes (cel/µL)	ln(x)
Total lymphocytes (cel/µL)	ln(x)
NK lymphocytes (%)	ln(x)
NK lymphocytes (cel/µL)	ln(x)
NKT lymphocytes (%)	ln(x)
NKT lymphocytes (cel/µL)	ln(x)
CD19 B lymphocytes (%)	log10(x)
CD19 B lymphocytes (cel/µL)	ln(x)
TCD3+ lymphocytes (%)	sqrt(x)
TCD3+ lymphocytes (cel/µL)	ln(x)
TCD4+CD62+ lymphocytes (cel/µL)	sqrt(x)
TCD4+CD62- lymphocytes (cel/µL)	sqrt(x)
TCD4+CD45RA+ lymphocytes (%)	ln(x)
TCD4+CD45RA+ lymphocytes (cel/µL)	ln(x)
TCD4+CD45RO+ lymphocytes (cel/µL)	ln(x)
TCD4+CD45RO+ lymphocytes (%)	x^2

TCD4+CD45RA+CD45RO+ lymphocytes (%)	ln(x)
TCD4+CD45RA+CD45RO+ lymphocytes (cel/µL)	1/sqrt(x)
TCD8+ lymphocytes (%)	1/sqrt(x)
TCD8+ lymphocytes (cel/µL)	1/sqrt(x)
TCD8+CD28+ lymphocytes (cel/µL)	ln(x)
TCD8+CD28- lymphocytes (cel/µL)	ln(x)
TCD8+CD45RA+ lymphocytes (cel/µL)	ln(x)
TCD8+CD45RO+ lymphocytes (cel/µL)	ln(x)
TCD8+CD45RA+CD45RO+ lymphocytes (cel/µL)	1/sqrt(x)
Abbreviations: BMI, body mass index; VF, Visceral Fat; FFM, fat free mass; HbA1c, glycated hemoglobin; HDL-c, high-density cholesterol; CRP, C-reactive protein; DBP, diastolic blood pressure; SBP, systolic blood pressure; NK, natural killer cells; NKT, natural killer T cells; CD3+, T cells; TCD4+, helper T cells; TCD4+CD62+, non-effector helper T cells; TCD4+CD62-, effector helper T cells, TCD4+CD45RA+: naive helper T cells; TCD4+CD45RO, memory helper T cell; TCD4+CD45RA+CD45RO+, in transition from naive to memory helper T cells; TCD8+, cytotoxic T cells; TCD8+CD28+, no activated cytotoxic T cells; TCD8+CD28-, activated cytotoxic T cells; TCD8+CD45RA+: naive cytotoxic T cells; TCD8+CD45RO+: memory cytotoxic T cell; TCD8+CD45RA+CD45RO+: in transition from naive to memory cytotoxic T cells. ln(x): natural logarithm; 1/sqrt(x): inverse of the square root, 1/(x^2): inverse-square; sqrt(x): square root; log10(x): logarithm with base 10; x^2: square; 1/x: inverse.	

5- The normally distributed data are presented as the mean and standard deviation of the mean, and the non-normally distributed variables are presented as the median and interquartile interval, to metabolic, body composition, and clinical characteristics and (Tables 7S) and immune cells relative and absolute values (Tables 8S & 9S respectively) according to the total body fat percentage. We used IBM SPSS Statistics Version 25.0 (USA).

Table 7.1S. **Descriptive Statistics Normal body fat [a]**

	TBF (%)	Weight (kg)	BMI (kg/m^2)	WC (cm)	FFM (kg)	FFM (%)	VF (cm2)	Glucose (mg/dL)	HbA1c (%)
Mean	22.56	62.11	22.25	78.53	48.24	77.43	72.75	81.08	5.41
Median	22.40	61.40	22.70	80.40	48.42	77.60	69.00	80.25	5.40
Desviation	4.44	12.91	2.716	8.81	10.92	4.44	26.09	6.90	0.23
25 quartile	18.75	49.72	19.62	69.72	37.25	73.40	43.00	77.63	5.30
50 quartile	22.40	61.40	22.70	80.40	48.42	77.60	69.00	80.25	5.40
75 quartile	26.60	70.45	24.18	84.50	57.03	81.25	95.00	86.75	5.55

[a]TBF diagnosis. 0= Normal body fat continue…

… continue Table 7.1S. **Descriptive Statistics Normal body fat[a]**

	Insulin (µUI/mL)	Total cholesterol (mg/dL)	HDL-c (mg/dL)	LDL-c (mg/dL)	Triglycerides (mg/dL)	CRP (mg/dL)	SBP (mmHg)	DBP (mmHg
Mean	5.90	164.52	52.84	86.16	110.78	0.166	106.77	70.69
Median	6.38	161.70	49.95	83.05	100.05	0.077	106.00	70.00
Desviation	2.01	27.96	12.37	21.20	42.19	0.195	11.84	8.32
25 quartile	4.28	144.00	44.60	69.50	81.88	0.042	100.00	64.50
50 quartile	6.38	161.70	49.95	83.05	100.05	0.082	107.00	70.00
75 quartile	7.24	175.60	60.63	98.63	141.00	0.230	117.50	76.25

[a]TBF diagnosis. 0= Normal body fat

Table 7.2S. **Descriptive Statistics Class 1 and 2 obesity[a]**

	TBF (%)	Weight (kg)	BMI (kg/m^2)	WC (cm)	FFM (kg)	FFM (%)	VF (cm2)	Glucose (mg/dL)	HbA1c (%)
Mean	34.56	77.87	28.74	97.42	51.13	65.44	128.28	97.76	7.40
Median	34.80	72.00	28.18	97.70	47.00	65.20	112.60	85.30	5.40
Desviation	3.95	22.29	5.71	14.61	15.61	3.95	59.46	63.10	4.00

25 quartile	31.70	63.85	24.68	88.35	41.96	61.60	98.55	75.50	5.30
50 quartile	34.80	72.00	28.18	97.70	47.00	65.20	112.60	85.30	5.40
75 quartile	38.40	82.90	29.85	102.25	54.72	68.30	137.75	95.20	10.50

[a]TBF diagnosis. 2= Class 1 and 2 obesity continue…

… continue Table 7.2S. **Descriptive Statistics Class 1 and 2 obesity[a]**

	Insulin (µUI/mL)	Total cholesterol (mg/dL)	HDL-c (mg/dL)	LDL-c (mg/dL)	Triglycerides (mg/dL)	CRP (mg/dL)	SBP (mmHg)	DBP (mmHg
Mean	20.04	171.97	45.57	93.46	163.52	0.646	112.72	74.80
Median	14.99	171.97	43.90	92.70	128.30	0.720	110.00	76.00
Desviation	11.17	35.80	13.27	24.10	101.52	0.315	11.02	10.63
25 quartile	11.36	141.85	34.55	76.00	107.55	0.357	107.50	67.00
50 quartile	14.99	171.97	43.90	92.70	128.30	0.726	110.00	76.00
75 quartile	31.25	199.50	53.40	117.75	184.45	0.883	120.00	80.00

TBF diagnosis. 0= Normal body fat

Table 7.3S. **Descriptive Statistics Class 3 obesity[a]**

	TBF (%)	Weight (kg)	BMI (kg/m^2)	WC (cm)	FFM (kg)	FFM (%)	VF (cm2)	Glucose (mg/dL)	HbA1c (%)
Mean	40.99	104.46	38.60	120.22	61.99	59.01	210.28	105.67	6.28
Median	41.90	94.50	37.38	112.90	55.78	58.10	173.00	99.00	5.80
Desviation	2.78	30.37	8.57	20.10	19.23	2.78	92.49	37.08	1.10
25 quartile	38.50	86.10	32.93	107.00	47.79	56.80	147.00	83.00	5.55
50 quartile	41.90	94.50	37.38	112.90	55.78	58.10	173.00	99.00	5.80
75 quartile	43.20	129.50	42.61	135.00	78.90	61.50	277.00	113.60	6.70

[a]TBF diagnosis. 3= Class 3 obesity continue…

… continue Table 7.3S. **Descriptive Statistics Class 3 obesity[a]**

	Insulin (µUI/mL)	Total cholesterol (mg/dL)	HDL-c (mg/dL)	LDL-c (mg/dL)	Triglycerides (mg/dL)	CRP (mg/dL)	SBP (mmHg)	DBP (mmHg
Mean	22.24	173.39	39.19	97.98	167.78	0.856	118.19	78.77
Median	22.49	174.00	37.00	98.80	138.80	0.600	120.00	80.00
Desviation	9.79	32.62	8.60	28.43	100.87	0.584	10.55	7.64
25 quartile	12.71	148.00	33.00	82.00	105.00	0.397	110.00	72.00
50 quartile	22.69	174.00	37.00	98.80	138.80	0.605	120.00	80.00
75 quartile	27.65	202.00	42.00	124.00	186.00	1.340	126.00	84.00

TBF diagnosis. 3= Class 3 obesity

Table 7.4S. **Descriptive Statistics Class 4 obesity[a]**

	TBF (%)	Weight (kg)	BMI (kg/m^2)	WC (cm)	FFM (kg)	FFM (%)	VF (cm2)	Glucose (mg/dL)	HbA1c (%)
Mean	50.70	117.18	44.45	129.30	57.55	49.30	254.07	104.51	5.94
Median	50.80	112.20	43.68	128.00	53.01	49.20	264.00	103.00	5.70
Desviation	4.77	24.60	6.76	16.02	12.85	4.77	66.16	15.48	0.94
25 quartile	47.28	101.50	39.58	116.75	48.59	47.05	199.00	95.00	5.50
50 quartile	50.80	112.20	43.68	128.00	53.01	49.20	264.00	103.00	5.70
75 quartile	52.95	137.55	50.15	139.25	60.61	52.73	290.00	111.00	6.00

[a]TBF diagnosis. 4= Class 4 obesity continue…

… continue Table 7.4S. **Descriptive Statistics Class 4 obesity[a]**

	Insulin (µUI/mL)	Total cholesterol (mg/dL)	HDL-c (mg/dL)	LDL-c (mg/dL)	Triglycerides (mg/dL)	CRP (mg/dL)	SBP (mmHg)	DBP (mmHg
Mean	23.84	175.20	43.33	103.18	155.63	1.073	122.90	76.79

Median	21.10	173.75	41.45	101.50	150.00	0.835	125.00	77.50
Desviation	13.14	31.32	9.65	27.89	66.88	0.985	13.60	11.47
25 quartile	14.46	156.00	36.75	80.75	106.75	0.439	110.00	70.00
50 quartile	21.10	173.75	41.45	101.50	150.00	0.840	125.00	77.50
75 quartile	30.35	192.73	48.10	121.50	195.25	1.155	132.00	82.25

TBF diagnosis. 4= Class 4 obesity

Table 8.1S. **Descriptive Statistics Normal body fat** [a]

(%)	Total lymphocytes	Monocytes	Granulocytes	TCD3+ lymphocytes	NK lymphocytes	NKT lymphocytes	B lymphocytes	TCD4+CD62+ lymphocytes	TCD4+CD62- lymphocytes (%)	TCD4+ lymphocytes
Mean	27.08	7.31	65.23	58.14	21.13	4.11	15.96	75.82	23.60	49.49
Median	25.77	7.57	65.10	59.22	22.47	3.91	14.64	80.60	19.60	51.55
Desviation	12.02	1.85	11.44	16.33	11.19	3.32	8.85	18.59	18.08	10.12
25 quartile	17.79	6.33	59.63	47.16	11.38	1.49	10.12	71.80	13.43	43.48
50 quartile	25.77	7.57	65.10	59.22	22.47	3.91	14.64	80.60	19.60	51.55
75 quartile	33.17	8.73	74.34	66.52	30.27	5.49	20.97	87.50	28.20	57.85

[a]TBF diagnosis. 0= Normal body fat continue…

… continue Table 8.1S. **Descriptive Statistics Normal body fat**[a]

(%)	TCD8+ lymphocytes	TCD8+CD28+ lymphocytes	TCD8+CD28- lymphocytes	TCD4+CD45RA+ lymphocytes	TCD4+CD45RA+CD45RO+ lymphocytes	TCD4+CD45RO+ lymphocytes	TCD8+CD45RA+ lymphocytes	TCD8+CD45RA+CD45RO+ lymphocytes	TCD8+CD45RO+ lymphocytes
Mean	51.99	46.12	47.64	24.71	10.41	62.48	44.98	16.51	35.89
Median	56.80	37.25	53.65	23.00	9.65	61.30	43.75	15.15	34.05
Desviation	21.08	21.46	23.68	13.74	3.07	13.18	11.92	9.46	11.11
25 quartile	31.70	26.95	24.85	13.55	8.28	55.83	33.78	10.60	28.30
50 quartile	56.80	37.25	53.65	23.00	9.65	61.30	43.75	15.15	34.05
75 quartile	65.88	63.78	67.78	32.50	12.88	73.93	56.93	20.00	46.58

[a]TBF diagnosis. 0= Normal body fat

Table 8.2S. **Descriptive Statistics Class 1 and 2 obesity**[a]

(%)	Total lymphocytes	Monocytes	Granulocytes	TCD3+ lymphocytes	NK lymphocytes	NKT lymphocytes	B lymphocytes	TCD4+CD62+ lymphocytes	TCD4+CD62- lymphocytes	TCD4+ lymphocytes
Mean	34.96	7.77	56.91	71.45	14.64	4.22	13.57	59.54	35.96	52.84
Median	35.00	7.20	60.10	77.50	12.60	2.94	9.89	62.50	35.00	53.10
Desviation	13.59	3.29	14.31	12.28	7.90	3.47	11.04	13.12	12.43	11.32
25 quartile	25.09	5.50	42.80	67.10	9.30	2.32	7.65	48.45	26.55	45.80
50 quartile	35.00	7.20	60.10	77.50	12.60	2.94	9.90	62.50	35.00	53.10
75 quartile	45.70	9.40	66.30	79.70	18.76	6.75	15.60	67.95	48.00	62.60

[a]TBF diagnosis. 2= Class 1 and 2 obesity continue…

… continue Table 8.2S. **Descriptive Statistics Class 1 and 2 obesity[a]**									
(%)	TCD8+ lymphocytes	TCD8+ CD28+ lymphocytes	TCD8+ CD28- lymphocytes	TCD4+CD45RA+ lymphocytes	TCD4+CD45RA+CD45RO+ lymphocytes	TCD4+CD45RO+ lymphocytes	TCD8+CD45RA+ lymphocytes	TCD8+CD45RA+CD45RO+ lymphocytes	TCD8+CD45RO+ lymphocytes
Mean	36.20	57.39	40.31	35.64	13.46	46.45	45.84	15.14	35.22
Median	36.10	59.00	35.95	32.40	13.25	42.05	46.20	13.75	36.10
Desviation	12.52	17.40	16.78	17.76	5.30	17.25	16.41	6.88	15.05
25 quartile	25.70	45.10	28.40	21.35	8.55	34.73	34.48	10.43	23.05
50 quartile	35.90	59.00	35.95	32.40	13.25	42.05	46.20	13.75	36.10
75 quartile	41.00	71.40	47.70	48.98	18.78	62.45	57.60	20.95	43.73
[a]TBF diagnosis. 2= Class 1 and 2 obesity									

Table 8.3S. **Descriptive Statistics Class 3 obesity[a]**										
(%)	Total lymphocytes	Monocytes	Granulocytes (%)	TCD3+ lymphocytes	NK lymphocytes	NKT lymphocytes	B lymphocytes	TCD4+CD62+ lymphocytes	TCD4+CD62- lymphocytes	TCD4+ lymphocytes
Mean	31.60	7.32	61.03	68.10	20.32	5.05	9.85	54.43	37.23	58.74
Median	30.14	6.71	62.20	73.22	19.51	2.57	9.68	54.30	37.20	53.10
Desviation	10.66	2.75	11.43	14.30	10.66	4.56	4.12	21.92	20.47	14.22
25 quartile	25.16	5.60	52.24	56.71	12.27	1.14	6.99	40.50	19.35	45.80
50 quartile	30.14	6.71	62.20	73.12	19.51	2.57	9.68	54.30	37.20	53.10
75 quartile	40.42	8.67	68.74	78.50	25.12	9.82	11.99	69.60	48.45	62.60
[a]TBF diagnosis. 3= Class 3 obesity continue…										

… continue Table 8.3S. **Descriptive Statistics Class 3 obesity[a]**									
(%)	TCD8+ lymphocytes	TCD8+ CD28+ lymphocytes	TCD8+ CD28- lymphocytes	TCD4+CD45RA+ lymphocytes	TCD4+CD45RA+CD45RO+ lymphocytes	TCD4+CD45RO+ lymphocytes	TCD8+CD45RA+ lymphocytes	TCD8+CD45RA+CD45RO+ lymphocytes	TCD8+CD45RO+ lymphocytes
Mean	38.66	49.64	45.61	24.93	14.03	59.07	34.97	15.98	43.70
Median	31.60	44.95	48.55	21.90	11.95	65.25	33.80	15.90	45.20
Desviation	20.05	18.19	19.18	14.37	10.16	26.77	14.97	9.08	19.76
25 quartile	26.80	36.13	32.00	14.95	7.18	41.43	22.60	9.70	26.65
50 quartile	31.60	44.95	48.55	21.90	11.95	65.05	33.80	15.90	45.20
75 quartile	43.04	62.88	60.10	32.00	16.98	74.73	44.98	24.53	64.60

[a]TBF diagnosis. 3= Class 3 obesity

Table 8.4S. **Descriptive Statistics Class 4 obesity**[a]										
(%)	Total lymphocytes	Monocytes	Granulocytes	TCD3+ lymphocytes	NK lymphocytes	NKT lymphocytes	B lymphocytes	TCD4+CD62+ lymphocytes	TCD4+CD62- lymphocytes	TCD4+ lymphocytes
Mean	29.15	7.61	63.24	68.39	19.31	4.06	9.06	54.85	44.00	59.74
Median	27.27	7.18	65.81	68.38	18.41	3.59	7.71	55.90	43.80	61.45
Desviation	11.47	3.09	11.45	11.91	9.19	2.99	5.95	18.61	18.72	8.81
25 quartile	21.34	5.71	55.48	60.74	11.95	1.65	6.46	45.20	29.15	54.00
50 quartile	27.27	7.18	65.81	68.38	18.41	3.59	7.71	55.90	43.80	61.45
75 quartile	36.14	9.52	70.60	79.13	25.78	5.30	12.62	68.60	53.15	66.45
[a]TBF diagnosis. 4= Class 4 obesity continue…										

… continue Table 8.4S. **Descriptive Statistics Class 4 obesity**[a]									
(%)	TCD8+ lymphocytes	TCD8+CD28+ lymphocytes	TCD8+CD28- lymphocytes	TCD4+CD45RA+ lymphocytes	TCD4+CD45RA+CD45RO+ lymphocytes	TCD4+CD45RO+ lymphocytes	TCD8+CD45RA+ lymphocytes	TCD8+CD45RA+CD45RO+ lymphocytes	TCD8+CD45RO+ lymphocytes
Mean	31.61	53.54	42.42	29.51	13.00	57.24	35.41	15.59	49.64
Median	29.00	54.10	44.05	24.35	12.80	65.05	34.90	14.70	46.50
Desviation	12.40	19.15	19.73	17.60	5.74	19.98	17.39	7.24	20.82
25 quartile	25.90	42.83	28.85	14.35	8.10	43.15	18.70	9.00	35.50
50 quartile	29.00	54.10	44.05	24.35	12.80	65.25	34.90	14.70	46.50
75 quartile	34.20	68.63	56.18	42.30	16.58	72.55	47.88	21.50	70.05
[a]TBF diagnosis. 4= Class 4 obesity continue…									

Table 9.1S. **Descriptive Statistics Normal body fat** [a]											
(cel/µL)	Leukocytes	Total lymphocytes	Monocytes	Granulocytes	TCD3+ lymphocytes	NK lymphocytes	NKT lymphocytes	B lymphocytes	TCD4+CD62+ lymphocytes	TCD4+CD62- lymphocytes	TCD4+ lymphocytes
Mean	5716.67	1266.26	419.53	4002.60	742.69	325.44	65.71	168.32	986.84	279.03	618.48
Median	5800.00	1188.72	419.53	4167.93	716.94	308.13	41.24	154.37	877.99	150.38	507.90
Desviation	1151.60	551.58	137.88	962.34	374.48	157.70	56.80	85.07	544.93	336.57	317.19
25 quartile	4900.00	749.08	332.99	3123.00	390.33	188.40	20.67	114.60	624.83	92.58	374.90
50 quartile	5800.00	1188.72	419.53	4167.93	716.94	308.13	41.24	154.37	877.99	150.38	507.90
75 quartile	6675.00	1725.08	488.36	4585.71	973.91	420.29	101.56	200.58	1478.57	401.15	904.33

[a]TBF diagnosis. 0= Normal body fat continue…

… continue Table 9.1S. **Descriptive Statistics Normal body fat**[a]									
(cel/μL)	TCD8+ lymphocytes	TCD8+ CD28+ lymphocytes	TCD8+ CD28- lymphocytes	TCD4+CD45RA+ lymphocytes	TCD4+CD45RA+CD45RO+ lymphocytes	TCD4+CD45RO+ lymphocytes	TCD8+CD45RA+ lymphocytes	TCD8+CD45RA+CD45RO+ lymphocytes	TCD8+CD45RO+ lymphocytes
Mean	734.98	552.11	604.77	342.72	130.73	772.29	577.26	226.09	448.46
Median	626.15	338.79	508.16	307.11	118.85	773.76	495.32	178.15	392.14
Desviation	362.35	462.01	429.10	250.91	73.70	327.28	310.63	143.00	253.97
25 quartile	493.08	249.62	251.71	131.34	69.72	434.80	373.43	112.09	246.56
50 quartile	626.15	338.79	508.16	307.11	118.85	773.76	495.32	178.15	392.14
75 quartile	859.81	795.00	875.30	602.50	141.41	1083.05	740.51	311.55	602.51
[a]TBF diagnosis. 0= Normal body fat									

Table 9.2S. **Descriptive Statistics Class 1 and 2 obesity**[a]											
(cel/μL)	Leukocytes	Total lymphocytes	Monocytes	Granulocytes	TCD3+ lymphocytes	NK lymphocytes	NKT lymphocytes	B lymphocytes	TCD4+CD62+ lymphocytes	TCD4+CD62- lymphocytes	TCD4+ lymphocytes
Mean	6761.67	1464.73	472.83	4680.27	960.14	295.33	55.39	195.56	836.00	546.50	805.06
Median	6800.00	1363.50	418.98	4886.02	952.39	182.78	48.43	172.59	840.20	413.73	804.27
Desviation	1240.65	711.35	152.81	842.48	438.74	308.12	38.05	93.23	467.86	409.02	296.84
25 quartile	6100.00	844.85	339.24	4080.51	543.66	145.93	20.46	125.42	393.24	330.82	525.99
50 quartile	6800.00	1363.50	418.98	4886.02	952.39	182.78	48.43	172.59	840.20	413.73	804.27
75 quartile	7642.50	2010.82	644.21	5214.18	1384.17	388.05	93.81	277.18	1283.97	723.46	1076.98
[a]TBF diagnosis. 2= Class 1 and 2 obesity continue…											

… continue Table 9.2S. **Descriptive Statistics Class 1 and 2 obesity**[a]									
(cel/μL)	TCD8+ lymphocytes	TCD8+ CD28+ lymphocytes	TCD8+ CD28- lymphocytes	TCD4+CD45RA+ lymphocytes	TCD4+CD45RA+CD45RO+ lymphocytes	TCD4+CD45RO+ lymphocytes	TCD8+CD45RA+ lymphocytes	TCD8+CD45RA+CD45RO+ lymphocytes	TCD8+CD45RO+ lymphocytes
Mean	556.52	819.27	593.31	540.77	122.49	801.17	549.54	273.26	642.06
Median	403.61	580.79	401.03	392.17	146.6	773.36	455.56	169.95	638.06
Desviation	454.11	464.17	462.48	480.13	73.18	460.80	420.19	260.60	249.28
25 quartile	222.12	515.74	247.38	196.26	102.2	414.56	218.87	76.56	438.82

50 quartile	403.61	580.79	401.03	392.17	198.12	773.36	455.56	169.95	638.06
75 quartile	866.66	1348.57	1146.52	839.69	485.63	1215.91	826.09	516.32	799.76
[a]TBF diagnosis. 2= Class 1 and 2 obesity									

Table 9.3S. **Descriptive Statistics Class 3 obesity[a]**

(cel/µL)	Leukocytes	Total lymphocytes	Monocytes	Granulocytes	TCD3+ lymphocytes	NK lymphocytes	NKT lymphocytes	B lymphocytes	TCD4+CD62+ lymphocytes	TCD4+CD62- lymphocytes	TCD4+ lymphocytes
Mean	8794.29	2680.90	635.73	5487.18	1788.73	575.01	125.92	248.92	1496.65	1140.52	805.06
Median	8200.00	2365.01	569.06	4349.86	1412.78	467.52	51.40	237.15	1120.19	844.54	1122.27
Desviation	3019.12	1340.09	324.51	2465.69	1196.64	353.72	153.73	133.20	1134.34	887.77	296.84
25 quartile	6400.00	1613.94	348.06	3804.67	863.12	397.03	27.01	115.88	730.34	598.34	825.99
50 quartile	8200.00	2365.01	569.06	4349.86	1412.78	467.52	51.40	237.15	1120.19	844.54	1004.27
75 quartile	10800.00	3630.83	889.08	6511.51	2484.41	726.53	174.50	383.93	2163.90	1350.99	2213.98
[a]TBF diagnosis. 3= Class 3 obesity								continue…			

… continue Table 9.3S. **Descriptive Statistics Class 3 obesity[a]**

(cel/µL)	TCD8+ lymphocytes (cel/µL)	TCD8+CD28+ lymphocytes (cel/µL)	TCD8+CD28- lymphocytes (cel/µL)	TCD4+CD45RA+ lymphocytes (cel/µL)	TCD4+CD45RA+CD45RO+ lymphocytes (cel/µL)	TCD4+CD45RO+ lymphocytes (cel/µL)	TCD8+CD45RA+ lymphocytes (cel/µL)	TCD8+CD45RA+CD45RO+ lymphocytes (cel/µL)	TCD8+CD45RO+ lymphocytes (cel/µL)
Mean	1185.50	1349.96	1154.46	534.75	389.08	1784.66	813.14	391.26	1325.69
Median	957.87	1075.79	955.27	459.81	209.47	1560.98	702.92	220.56	931.68
Desviation	1178.05	895.14	797.15	396.86	586.21	886.93	439.55	431.42	542.33
25 quartile	439.27	686.43	505.19	294.78	131.95	1219.34	487.50	172.65	736.83
50 quartile	957.87	1075.79	955.27	459.81	202.47	1560.98	702.92	220.56	1173.68
75 quartile	1160.99	1843.98	1660.13	598.28	504.3	2497.02	1107.13	381.49	1587.88
[a]TBF diagnosis. 3= Class 3 obesity									

Table 9.4S. **Descriptive Statistics Class 4 obesity[a]**

(cel/µL)	Leukocytes	Total lymphocytes	Monocytes	Granulocytes	TCD3+ lymphocytes	NK lymphocytes	NKT lymphocytes	B lymphocytes	TCD4+CD62+	TCD4+CD62-	TCD4+ lymphocytes

									lymphocytes	lymphocytes	
Mean	7942.23	2250.28	729.72	5054.64	1566.83	428.84	76.99	218.72	1256.82	989.47	1344.09
Median	7965.00	2014.92	584.50	4552.36	1438.90	332.14	62.95	205.75	1164.84	873.42	1288.31
Desviation	2614.58	1090.39	686.75	1909.30	817.84	388.69	84.25	126.63	731.25	611.70	608.18
25 quartile	5825.00	1498.42	383.77	3602.72	1058.94	223.44	26.96	120.86	694.48	667.43	931.43
50 quartile	7965.00	2014.92	584.50	4552.36	1438.90	332.14	62.95	165.75	1164.84	773.42	1288.31
75 quartile	9825.00	2704.09	797.01	6548.67	2091.19	475.77	103.31	350.05	1883.11	1308.46	1650.17
[a]TBF diagnosis. 4= Class 4 obesity continue…											

… continue Table 9.4S. **Descriptive Statistics Class 4 obesity**[a]									
(cel/µL)	TCD8+ lymphocytes	TCD8+CD28+ lymphocytes	TCD8+CD28- lymphocytes	TCD4+CD45RA+ lymphocytes	TCD4+CD45RA+CD45RO+ lymphocytes	TCD4+CD45RO+ lymphocytes	TCD8+CD45RA+ lymphocytes	TCD8+CD45RA+CD45RO+ lymphocytes	TCD8+CD45RO+ lymphocytes
Mean	704.15	1247.85	871.63	688.99	319.72	1324.95	967.02	382.86	1171.42
Median	624.93	1016.05	798.29	539.34	224.83	1220.99	1173.09	268.39	931.74
Desviation	414.85	846.72	488.14	542.64	247.16	725.85	650.55	262.71	672.44
25 quartile	416.10	572.78	565.67	256.99	153.48	707.72	713.08	178.04	736.48
50 quartile	624.93	1016.05	798.29	539.34	224.83	1220.99	702.09	268.39	931.74
75 quartile	834.47	1656.59	1075.30	1118.87	491.56	1785.65	1706.20	580.30	1587.19
[a]TBF diagnosis. 4= Class 4 obesity									

6- We applied one-way analysis of variance (ANOVA) to each variable to estimate the difference among the groups. We considered $p < 0.05$ to indicate a statistically significant difference. To metabolic, body composition, and clinical characteristics and (Tables 10S) and immune cells relative and absolute values (Tables 11S & 12S respectively) according to the total body fat percentage. We used IBM SPSS Statistics Version 25.0 (USA).

Table 10S. **ANOVA one-way.** Metabolic, body composition, and clinical variables						
		sum of squares	gl	quare root of the arithmetic mean	F	Sig.
Weight (kg)	between groups	58854.236	3	19618.079	34.227	0.000
	within groups	68780.419	120	573.170		
	Total	127634.655	123			
WC (cm)	between groups	48736.918	3	16245.639	65.612	0.000
	within groups	29712.171	120	247.601		
	Total	78449.088	123			
TBF (%)	between groups	13389.863	3	4463.288	263.322	0.000
	within groups	2033.994	120	16.950		
	Total	15423.857	123			
FFM (%)	between groups	13389.863	3	4463.288	263.322	0.000

	within groups	2033.994	120	16.950		
	Total	15423.857	123			
Total cholesterol (mg/dL)	between groups	1939.048	3	646.349	0.634	0.595
	within groups	122342.888	120	1019.524		
	Total	124281.935	123			
LDL-c (mg/dL)	between groups	4951.090	3	1650.363	2.429	0.069
	within groups	81548.020	120	679.567		
	Total	86499.110	123			
BMI (kg/m2)	between groups	8.758	3	2.919	105.828	0.000
	within groups	3.310	120	0.028		
	Total	12.068	123			
FFM (kg)	between groups	0.005	3	0.002	5.535	0.001
	within groups	0.034	120	0.000		
	Total	0.039	123			
Glucose (mg/dL)	between groups	0.000	3	0.000	11.496	0.000
	within groups	0.000	120	0.000		
	Total	0.000	123			
HDL-c (mg/dL)	between groups	0.007	3	0.002	8.107	0.000
	within groups	0.035	120	0.000		
	Total	0.042	123			
Triglycerides (mg/dL)	between groups	0.005	3	0.002	4.319	0.006
	within groups	0.045	120	0.000		
	Total	0.050	123			
Insulin (µUI/mL)	between groups	69.738	3	23.246	19.210	0.000
	within groups	96.809	80	1.210		
	Total	166.547	83			
CRP (mg/dL)	between groups	4.369	3	1.456	11.834	0.000
	within groups	9.846	80	0.123		
	Total	14.215	83			
VF (cm2)	between groups	29.850	3	9.950	81.522	0.000
	within groups	14.646	120	0.122		
	Total	44.496	123			
HbA1c (mg/dL)	between groups	0.009	3	0.003	19.087	0.000
	within groups	0.019	120	0.000		
	Total	0.028	123			
SBP (mmHg)	between groups	0.352	3	0.117	10.884	0.000
	within groups	1.292	120	0.011		
	Total	1.644	123			
DBP (mmHg)	between groups	3.400	3	1.133	3.523	0.017
	within groups	38.600	120	0.322		
	Total	42.000	123			

Table 11S. **ANOVA one-way. immune cells relative values**

		sum of squares	gl	quare root of the arithmetic mean	F	Sig.
Total lymphocytes (%)	between groups	874.918	3	291.639	2.089	0.105
	within groups	16475.461	118	139.623		
	Total	17350.379	121			
Monocytes (%)	between groups	4.108	3	1.369	0.171	0.916
	within groups	943.004	118	7.992		
	Total	947.112	121			
Granulocytes (%)	between groups	959.226	3	319.742	2.211	0.090
	within groups	17066.420	118	144.631		
	Total	18025.647	121			
TCD4+ lymphocytes (%)	between groups	2153.114	3	717.705	5.762	0.001
	within groups	14699.042	118	124.568		
	Total	16852.156	121			

TCD4+CD62+ lymphocytes (%)	between groups	8518.727	3	2839.576	8.001	0.000
	within groups	39038.704	110	354.897		
	Total	47557.431	113			
TCD4+CD62- lymphocytes (%)	between groups	6767.122	3	2255.707	6.751	0.000
	within groups	36754.488	110	334.132		
	Total	43521.610	113			
TCD8+CD28+ lymphocytes (%)	between groups	1779.259	3	593.086	1.612	0.191
	within groups	38627.884	105	367.885		
	Total	40407.143	108			
TCD8+CD28- lymphocytes (%)	between groups	811.113	3	270.371	0.672	0.571
	within groups	44231.493	110	402.104		
	Total	45042.606	113			
TCD8+CD45RA+ lymphocytes (%)	between groups	2379.067	3	793.022	3.203	0.027
	within groups	22284.707	90	247.608		
	Total	24663.774	93			
TCD8+CD45RO+ lymphocytes (%)	between groups	3642.137	3	1214.046	3.683	0.015
	within groups	31313.207	95	329.613		
	Total	34955.344	98			
NK lymphocytes (%)	between groups	1.687	3	0.562	1.787	0.154
	within groups	35.875	114	0.315		
	Total	37.562	117			
NKT lymphocytes (%)	between groups	0.183	3	0.061	0.053	0.984
	within groups	73.259	64	1.145		
	Total	73.442	67			
TCD8+ lymphocytes (%)	between groups	0.022	3	0.007	6.221	0.001
	within groups	0.134	113	0.001		
	Total	0.156	116			
TCD4+CD45RA+ lymphocytes (%)	between groups	1.976	3	0.659	1.804	0.152
	within groups	33.603	92	0.365		
	Total	35.579	95			
TCD4+CD45RA+CD45RO + lymphocytes (%)	between groups	0.497	3	0.166	0.753	0.523
	within groups	20.689	94	0.220		
	Total	21.185	97			
TCD4+CD45RO+ lymphocytes (%)	between groups	36805775.943	3	12268591.981	2.016	0.117
	within groups	584301614.81	96	6086475.154		
	Total	621107390.75	99			
TCD8+CD45RA+CD45RO + lymphocytes (%)	between groups	0.223	3	0.074	0.280	0.840
	within groups	24.977	94	0.266		
	Total	25.200	97			
TCD3+ lymphocytes (%)	between groups	10.872	3	3.624	1.918	0.130
	within groups	226.730	120	1.889		
	Total	237.602	123			
B lymphocytes (%)	between groups	1.108	3	0.369	3.152	0.028
	within groups	14.063	120	0.117		
	Total	15.171	123			

Table 12S. **ANOVA one-way. immune cells absolute values**

		sum of squares	gl	quare root of the arithmetic mean	F	Sig.
Leukocytes (cel/µL)	between groups	1.617	3	0.539	3.807	0.013
	within groups	11.467	81	0.142		
	Total	13.084	84			
Total lymphocytes (cel/µL)	between groups	6.235	3	2.078	8.131	0.000
	within groups	20.960	82	0.256		

	Total	27.196	85			
Monocytes (cel/µL)	between groups	0.001	3	0.000	1.887	0.138
	within groups	0.013	82	0.000		
	Total	0.014	85			
Granulocytes (cel/µL)	between groups	0.700	3	0.233	1.380	0.255
	within groups	13.853	82	0.169		
	Total	14.553	85			
TCD3+ lymphocytes (cel/µL)	between groups	8.237	3	2.746	8.132	0.000
	within groups	27.351	81	0.338		
	Total	35.588	84			
NK lymphocytes (cel/µL)	between groups	3.841	3	1.280	2.996	0.036
	within groups	34.192	80	0.427		
	Total	38.033	83			
NKT lymphocytes (cel/µL)	between groups	1.036	3	0.345	0.322	0.809
	within groups	67.466	63	1.071		
	Total	68.502	66			
B lymphocytes (cel/µL)	between groups	1.566	3	0.522	1.215	0.310
	within groups	33.937	79	0.430		
	Total	35.504	82			
TCD4+ lymphocytes (cel/µL)	between groups	9.773	3	3.258	12.520	0.000
	within groups	21.338	82	0.260		
	Total	31.111	85			
TCD4+CD62+ lymphocytes (cel/µL)	between groups	536.339	3	178.780	1.368	0.258
	within groups	10717.475	82	130.701		
	Total	11253.815	85			
TCD4+CD62- lymphocytes (cel/µL)	between groups	3678.795	3	1226.265	14.454	0.000
	within groups	6956.820	82	84.839		
	Total	10635.614	85			
TCD8+ lymphocytes (cel/µL)	between groups	0.001	3	0.000	2.813	0.045
	within groups	0.010	77	0.000		
	Total	0.011	80			
TCD8+CD28+ lymphocytes (cel/µL)	between groups	11.213	3	3.738	7.907	0.000
	within groups	33.563	71	0.473		
	Total	44.776	74			
TCD8+CD28- lymphocytes (cel/µL)	between groups	4.911	3	1.637	2.840	0.044
	within groups	40.352	70	0.576		
	Total	45.263	73			
TCD4+CD45RA+ lymphocytes (cel/µL)	between groups	7.432	3	2.477	3.966	0.011
	within groups	46.857	75	0.625		
	Total	54.289	78			
TCD4+CD45RA+CD45RO+ lymphocytes (cel/µL)	between groups	0.015	3	0.005	9.033	0.000
	within groups	0.041	75	0.001		
	Total	0.056	78			
TCD4+CD45RO+ lymphocytes (cel/µL)	between groups	7.055	3	2.352	6.740	0.000
	within groups	26.519	76	0.349		
	Total	33.574	79			
TCD8+CD45RA+ lymphocytes (cel/µL)	between groups	2.151	3	0.717	1.602	0.196
	within groups	32.221	72	0.448		
	Total	34.372	75			
TCD8+CD45RA+CD45RO+ lymphocytes (cel/µL)	between groups	0.005	3	0.002	2.939	0.039
	within groups	0.041	72	0.001		
	Total	0.046	75			
TCD8+CD45RO+ lymphocytes (cel/µL)	between groups	13.608	3	4.536	13.331	0.000
	within groups	24.839	73	0.340		
	Total	38.448	76			

7- We applied two-way ANOVA to each variable to estimate the difference among the groups by sex. We considered $p < 0.05$ to indicate a statistically significant difference. To metabolic, body composition, and clinical characteristics and immune cells relative and absolute values (Tables 13S) according to the total body fat percentage. We used IBM SPSS Statistics Version 25.0 (USA).

Table 13. two-way ANOVA. Metabolic, body composition, and clinical variables

Dependent variable	Factors: Diagnosis by TBF[a] and sex[b]
	Sig.[c]
TBF (%)	<0.001
Weight (kg)	<0.001
BMI (kg/m^2)	<0.001
WC (cm)	<0.001
FFM (kg)	<0.001
FFM (%)	<0.001
VF (cm^2)	<0.001
Glucose (mg/dL)	<0.001
HbA1c (%)	0.040
Insulin (µUI/mL)	<0.001
Total cholesterol (mg/dL)	0.080
HDL-c (mg/dL)	<0.001
LDL-c (mg/dL)	0.027
Triglycerides (mg/dL)	<0.001
CRP (mg/dL)	<0.001
SBP (mmHg)	<0.001
DBP (mmHg)	0.002
Leukocytes (%)	0.001
Monocytes (%)	0.233
Monocytes (cel/µL)	0.003
Granulocytes (%)	0.337
Granulocytes (cel/µL)	0.097
Total lymphocytes (%)	0.730
Total lymphocytes (cel/µL)	<0.001
NK (%)	0.618
NK (cel/µL)	0.086
NKT (%)	0.836
NKT (cel/µL)	0.357
CD19+ (%)	0.010
CD19+ (cel/µL)	0.043
CD3+ (%)	0.063
CD3+ (cel/µL)	<0.001
CD4+ (%)	<0.001
CD4+ (cel/µL)	<0.001
CD4+CD62- (%)	<0.001
CD4+CD62- (cel/µL)	<0.001
CD4+CD62+ (%)	<0.001
CD4+CD62+ (cel/µL)	0.009
CD4+CD45RA+ (%)	0.394
CD4+CD45RA+ (cel/µL)	0.002
CD4+CD45RO+ (%)	0.566
CD4+CD45RO+ (cel/µL)	<0.001
CD4+CD45RA+CD45RO+ (%)	0.324
CD4+CD45RA+CD45RO+ (cel/µL)	<0.001
CD8+ (%)	<0.001
CD8+ (cel/µL)	0.015
CD8+CD28- (%)	0.275
CD8+CD28- (cel/µL)	0.015

CD8+CD28+ (%)	0.196
CD8+CD28+ (cel/µL)	<0.001
CD8+CD45RA+ (%)	0.018
CD8+CD45RA+ (cel/µL)	0.066
CD8+CD45RO+ (%)	0.004
CD8+CD45RO+ (cel/µL)	<0.001
CD8+CD45RA+CD45RO+ (%)	0.794
CD8+CD45RA+CD45RO+ (cel/µL)	0.011

a. 1= Normal body fat, 2= Class 1 and 2 obesity, 3= Class 3 obesity, 4= Class 4 obesity
b. 0= male, 1= female
c. p@ = statistical significance

8- We used the Bonferroni test post hoc test to determine intergroup mean differences. We considered $p < 0.05$ to indicate a statistically significant difference. To metabolic, body composition, and clinical characteristics and (Tables 14S) and immune cells relative and absolute values (Tables 15S & 16S respectively) according to the total body fat percentage. We used IBM SPSS Statistics Version 25.0 (USA).

Table 14S. **Bonferroni test post hoc. Metabolic, body composition, and clinical variables**

ANOVA, multiple comparisons

			difference of means (I-J)	Desv. Error	Sig.	Confidence interval 95%	
						Lower limit	Upper limit
Weight (kg)	Normal body fat	Class 1 and 2 obesity	-15.74877	6.70610	0.123	-33.7392	2.2417
		Class 3 obesity	-42.34206*	6.36666	0.000	-59.4219	-25.2622
		Class 4 obesity	-55.05934*	5.97427	0.000	-71.0865	-39.0322
	Class 1 and 2 obesity	Normal body fat	15.74877	6.70610	0.123	-2.2417	33.7392
		Class 3 obesity	-26.59329*	6.43554	0.000	-43.8579	-9.3287
		Class 4 obesity	-39.31057*	6.04762	0.000	-55.5345	-23.0866
	Class 3 obesity	Normal body fat	42.34206*	6.36666	0.000	25.2622	59.4219
		Class 1 and 2 obesity	26.59329*	6.43554	0.000	9.3287	43.8579
		Class 4 obesity	-12.71728	5.66889	0.160	-27.9252	2.4906
	Class 4 obesity	Normal body fat	55.05934*	5.97427	0.000	39.0322	71.0865
		Class 1 and 2 obesity	39.31057*	6.04762	0.000	23.0866	55.5345
		Class 3 obesity	12.71728	5.66889	0.160	-2.4906	27.9252
WC (cm)	Normal body fat	Class 1 and 2 obesity	-18.88538*	4.40763	0.000	-30.7097	-7.0610
		Class 3 obesity	-41.68151*	4.18453	0.000	-52.9073	-30.4557
		Class 4 obesity	-50.86300*	3.92663	0.000	-61.3970	-40.3290
	Class 1 and 2 obesity	Normal body fat	18.88538*	4.40763	0.000	7.0610	30.7097
		Class 3 obesity	-22.79613*	4.22980	0.000	-34.1434	-11.4489
		Class 4 obesity	-31.97762*	3.97484	0.000	-42.6409	-21.3143
	Class 3 obesity	Normal body fat	41.68151*	4.18453	0.000	30.4557	52.9073
		Class 1 and 2 obesity	22.79613*	4.22980	0.000	11.4489	34.1434
		Class 4 obesity	-9.18149	3.72591	0.091	-19.1770	0.8140
	Class 4 obesity	Normal body fat	50.86300*	3.92663	0.000	40.3290	61.3970
		Class 1 and 2 obesity	31.97762*	3.97484	0.000	21.3143	42.6409
		Class 3 obesity	9.18149	3.72591	0.091	-0.8140	19.1770
TBF (%)	Normal body fat	Class 1 and 2 obesity	-11.99862*	1.15322	0.000	-15.0924	-8.9049
		Class 3 obesity	-18.42816*	1.09485	0.000	-21.3653	-15.4910
		Class 4 obesity	-28.13938*	1.02737	0.000	-30.8955	-25.3832
	Class 1 and 2 obesity	Normal body fat	11.99862*	1.15322	0.000	8.9049	15.0924
		Class 3 obesity	-6.42955*	1.10669	0.000	-9.3985	-3.4606
		Class 4 obesity	-16.14076*	1.03998	0.000	-18.9307	-13.3508
	Class 3 obesity	Normal body fat	18.42816*	1.09485	0.000	15.4910	21.3653
		Class 1 and 2 obesity	6.42955*	1.10669	0.000	3.4606	9.3985
		Class 4 obesity	-9.71121*	0.97486	0.000	-12.3265	-7.0960
	Class 4 obesity	Normal body fat	28.13938*	1.02737	0.000	25.3832	30.8955
		Class 1 and 2 obesity	16.14076*	1.03998	0.000	13.3508	18.9307

		Class 3 obesity	9.71121*	0.97486	0.000	7.0960	12.3265
FFM (%)	Normal body fat	Class 1 and 2 obesity	11.99862*	1.15322	0.000	8.9049	15.0924
		Class 3 obesity	18.42816*	1.09485	0.000	15.4910	21.3653
		Class 4 obesity	28.13938*	1.02737	0.000	25.3832	30.8955
	Class 1 and 2 obesity	Normal body fat	-11.99862*	1.15322	0.000	-15.0924	-8.9049
		Class 3 obesity	6.42955*	1.10669	0.000	3.4606	9.3985
		Class 4 obesity	16.14076*	1.03998	0.000	13.3508	18.9307
	Class 3 obesity	Normal body fat	-18.42816*	1.09485	0.000	-21.3653	-15.4910
		Class 1 and 2 obesity	-6.42955*	1.10669	0.000	-9.3985	-3.4606
		Class 4 obesity	9.71121*	0.97486	0.000	7.0960	12.3265
	Class 4 obesity	Normal body fat	-28.13938*	1.02737	0.000	-30.8955	-25.3832
		Class 1 and 2 obesity	-16.14076*	1.03998	0.000	-18.9307	-13.3508
		Class 3 obesity	-9.71121*	0.97486	0.000	-12.3265	-7.0960
Total cholesterol (mg/dL)	Normal body fat	Class 1 and 2 obesity	-7.462	8.944	1.000	-31.46	16.53
		Class 3 obesity	-8.849	8.491	1.000	-31.63	13.93
		Class 4 obesity	-10.676	7.968	1.000	-32.05	10.70
	Class 1 and 2 obesity	Normal body fat	7.462	8.944	1.000	-16.53	31.46
		Class 3 obesity	-1.387	8.583	1.000	-24.41	21.64
		Class 4 obesity	-3.214	8.066	1.000	-24.85	18.42
	Class 3 obesity	Normal body fat	8.849	8.491	1.000	-13.93	31.63
		Class 1 and 2 obesity	1.387	8.583	1.000	-21.64	24.41
		Class 4 obesity	-1.827	7.561	1.000	-22.11	18.46
	Class 4 obesity	Normal body fat	10.676	7.968	1.000	-10.70	32.05
		Class 1 and 2 obesity	3.214	8.066	1.000	-18.42	24.85
		Class 3 obesity	1.827	7.561	1.000	-18.46	22.11
LDL-c (mg/dL)	Normal body fat	Class 1 and 2 obesity	-7.30631	7.30204	1.000	-26.8955	12.2829
		Class 3 obesity	-11.81973	6.93244	0.545	-30.4174	6.7779
		Class 4 obesity	-17.02326	6.50518	0.060	-34.4747	0.4282
	Class 1 and 2 obesity	Normal body fat	7.30631	7.30204	1.000	-12.2829	26.8955
		Class 3 obesity	-4.51342	7.00744	1.000	-23.3123	14.2854
		Class 4 obesity	-9.71695	6.58505	0.856	-27.3827	7.9487
	Class 3 obesity	Normal body fat	11.81973	6.93244	0.545	-6.7779	30.4174
		Class 1 and 2 obesity	4.51342	7.00744	1.000	-14.2854	23.3123
		Class 4 obesity	-5.20353	6.17266	1.000	-21.7629	11.3558
	Class 4 obesity	Normal body fat	17.02326	6.50518	0.060	-0.4282	34.4747
		Class 1 and 2 obesity	9.71695	6.58505	0.856	-7.9487	27.3827
		Class 3 obesity	5.20353	6.17266	1.000	-11.3558	21.7629
BMI (kg/m2)	Normal body fat	Class 1 and 2 obesity	-.24693*	0.04652	0.000	-0.3717	-0.1221
		Class 3 obesity	-.53620*	0.04417	0.000	-0.6547	-0.4177
		Class 4 obesity	-.68750*	0.04145	0.000	-0.7987	-0.5763
	Class 1 and 2 obesity	Normal body fat	.24693*	0.04652	0.000	0.1221	0.3717
		Class 3 obesity	-.28927*	0.04465	0.000	-0.4090	-0.1695
		Class 4 obesity	-.44056*	0.04195	0.000	-0.5531	-0.3280
	Class 3 obesity	Normal body fat	.53620*	0.04417	0.000	0.4177	0.6547
		Class 1 and 2 obesity	.28927*	0.04465	0.000	0.1695	0.4090
		Class 4 obesity	-.15129*	0.03933	0.001	-0.2568	-0.0458
	Class 4 obesity	Normal body fat	.68750*	0.04145	0.000	0.5763	0.7987
		Class 1 and 2 obesity	.44056*	0.04195	0.000	0.3280	0.5531
		Class 3 obesity	.15129*	0.03933	0.001	0.0458	0.2568
FFM (kg)	Normal body fat	Class 1 and 2 obesity	0.00324	0.00474	1.000	-0.0095	0.0159
		Class 3 obesity	.01526*	0.00450	0.006	0.0032	0.0273
		Class 4 obesity	.01279*	0.00422	0.018	0.0015	0.0241
	Class 1 and 2 obesity	Normal body fat	-0.00324	0.00474	1.000	-0.0159	0.0095
		Class 3 obesity	0.01203	0.00455	0.056	-0.0002	0.0242
		Class 4 obesity	0.00956	0.00427	0.163	-0.0019	0.0210
	Class 3 obesity	Normal body fat	-.01526*	0.00450	0.006	-0.0273	-0.0032
		Class 1 and 2 obesity	-0.01203	0.00455	0.056	-0.0242	0.0002
		Class 4 obesity	-0.00247	0.00401	1.000	-0.0132	0.0083

	Class 4 obesity	Normal body fat	-.01279*	0.00422	0.018	-0.0241	-0.0015
		Class 1 and 2 obesity	-0.00956	0.00427	0.163	-0.0210	0.0019
		Class 3 obesity	0.00247	0.00401	1.000	-0.0083	0.0132
Glucose (mg/dL)	Normal body fat	Class 1 and 2 obesity	0.00001	0.00001	1.000	0.0000	0.0000
		Class 3 obesity	.00004*	0.00001	0.004	0.0000	0.0001
		Class 4 obesity	.00006*	0.00001	0.000	0.0000	0.0001
	Class 1 and 2 obesity	Normal body fat	-0.00001	0.00001	1.000	0.0000	0.0000
		Class 3 obesity	0.00003	0.00001	0.058	0.0000	0.0001
		Class 4 obesity	.00005*	0.00001	0.000	0.0000	0.0001
	Class 3 obesity	Normal body fat	-.00004*	0.00001	0.004	-0.0001	0.0000
		Class 1 and 2 obesity	-0.00003	0.00001	0.058	-0.0001	0.0000
		Class 4 obesity	0.00002	0.00001	0.810	0.0000	0.0000
	Class 4 obesity	Normal body fat	-.00006*	0.00001	0.000	-0.0001	0.0000
		Class 1 and 2 obesity	-.00005*	0.00001	0.000	-0.0001	0.0000
		Class 3 obesity	-0.00002	0.00001	0.810	0.0000	0.0000
HDL-c (mg/dL)	Normal body fat	Class 1 and 2 obesity	-0.01240	0.00478	0.064	-0.0252	0.0004
		Class 3 obesity	-.02217*	0.00454	0.000	-0.0343	-0.0100
		Class 4 obesity	-.01439*	0.00426	0.006	-0.0258	-0.0030
	Class 1 and 2 obesity	Normal body fat	0.01240	0.00478	0.064	-0.0004	0.0252
		Class 3 obesity	-0.00977	0.00459	0.212	-0.0221	0.0025
		Class 4 obesity	-0.00199	0.00431	1.000	-0.0136	0.0096
	Class 3 obesity	Normal body fat	.02217*	0.00454	0.000	0.0100	0.0343
		Class 1 and 2 obesity	0.00977	0.00459	0.212	-0.0025	0.0221
		Class 4 obesity	0.00778	0.00404	0.340	-0.0031	0.0186
	Class 4 obesity	Normal body fat	.01439*	0.00426	0.006	0.0030	0.0258
		Class 1 and 2 obesity	0.00199	0.00431	1.000	-0.0096	0.0136
		Class 3 obesity	-0.00778	0.00404	0.340	-0.0186	0.0031
Triglycerides (mg/dL)	Normal body fat	Class 1 and 2 obesity	.01473*	0.00544	0.047	0.0001	0.0293
		Class 3 obesity	.01611*	0.00517	0.014	0.0022	0.0300
		Class 4 obesity	.01525*	0.00485	0.013	0.0022	0.0283
	Class 1 and 2 obesity	Normal body fat	-.01473*	0.00544	0.047	-0.0293	-0.0001
		Class 3 obesity	0.00138	0.00522	1.000	-0.0126	0.0154
		Class 4 obesity	0.00052	0.00491	1.000	-0.0126	0.0137
	Class 3 obesity	Normal body fat	-.01611*	0.00517	0.014	-0.0300	-0.0022
		Class 1 and 2 obesity	-0.00138	0.00522	1.000	-0.0154	0.0126
		Class 4 obesity	-0.00086	0.00460	1.000	-0.0132	0.0115
	Class 4 obesity	Normal body fat	-.01525*	0.00485	0.013	-0.0283	-0.0022
		Class 1 and 2 obesity	-0.00052	0.00491	1.000	-0.0137	0.0126
		Class 3 obesity	0.00086	0.00460	1.000	-0.0115	0.0132
Insulin (µUI/mL)	Normal body fat	Class 1 and 2 obesity	-1.94955*	0.55965	0.005	-3.4637	-0.4355
		Class 3 obesity	-2.21382*	0.35890	0.000	-3.1848	-1.2428
		Class 4 obesity	-2.31679*	0.31733	0.000	-3.1753	-1.4583
	Class 1 and 2 obesity	Normal body fat	1.94955*	0.55965	0.005	0.4355	3.4637
		Class 3 obesity	-0.26427	0.54740	1.000	-1.7452	1.2167
		Class 4 obesity	-0.36724	0.52109	1.000	-1.7770	1.0426
	Class 3 obesity	Normal body fat	2.21382*	0.35890	0.000	1.2428	3.1848
		Class 1 and 2 obesity	0.26427	0.54740	1.000	-1.2167	1.7452
		Class 4 obesity	-0.10297	0.29519	1.000	-0.9016	0.6957
	Class 4 obesity	Normal body fat	2.31679*	0.31733	0.000	1.4583	3.1753
		Class 1 and 2 obesity	0.36724	0.52109	1.000	-1.0426	1.7770
		Class 3 obesity	0.10297	0.29519	1.000	-0.6957	0.9016
CRP (mg/dL)	Normal body fat	Class 1 and 2 obesity	-0.41818	0.17848	0.130	-0.9010	0.0647
		Class 3 obesity	-.51345*	0.11445	0.000	-0.8231	-0.2038
		Class 4 obesity	-.59325*	0.10120	0.000	-0.8670	-0.3195
	Class 1 and 2 obesity	Normal body fat	0.41818	0.17848	0.130	-0.0647	0.9010
		Class 3 obesity	-0.09527	0.17457	1.000	-0.5676	0.3770
		Class 4 obesity	-0.17507	0.16618	1.000	-0.6247	0.2745
	Class 3 obesity	Normal body fat	.51345*	0.11445	0.000	0.2038	0.8231

		Class 1 and 2 obesity	0.09527	0.17457	1.000	-0.3770	0.5676
		Class 4 obesity	-0.07980	0.09414	1.000	-0.3345	0.1749
	Class 4 obesity	Normal body fat	.59325*	0.10120	0.000	0.3195	0.8670
		Class 1 and 2 obesity	0.17507	0.16618	1.000	-0.2745	0.6247
		Class 3 obesity	0.07980	0.09414	1.000	-0.1749	0.3345
VF (cm2)	Normal body fat	Class 1 and 2 obesity	-.56020*	0.09786	0.000	-0.8227	-0.2977
		Class 3 obesity	-1.04587*	0.09291	0.000	-1.2951	-0.7966
		Class 4 obesity	-1.28576*	0.08718	0.000	-1.5196	-1.0519
	Class 1 and 2 obesity	Normal body fat	.56020*	0.09786	0.000	0.2977	0.8227
		Class 3 obesity	-.48568*	0.09391	0.000	-0.7376	-0.2337
		Class 4 obesity	-.72557*	0.08825	0.000	-0.9623	-0.4888
	Class 3 obesity	Normal body fat	1.04587*	0.09291	0.000	0.7966	1.2951
		Class 1 and 2 obesity	.48568*	0.09391	0.000	0.2337	0.7376
		Class 4 obesity	-.23989*	0.08272	0.027	-0.4618	-0.0180
	Class 4 obesity	Normal body fat	1.28576*	0.08718	0.000	1.0519	1.5196
		Class 1 and 2 obesity	.72557*	0.08825	0.000	0.4888	0.9623
		Class 3 obesity	.23989*	0.08272	0.027	0.0180	0.4618
HbA1c (mg/dL)	Normal body fat	Class 1 and 2 obesity	.01713*	0.00349	0.000	0.0078	0.0265
		Class 3 obesity	0.00407	0.00331	1.000	-0.0048	0.0130
		Class 4 obesity	-0.00637	0.00311	0.256	-0.0147	0.0020
	Class 1 and 2 obesity	Normal body fat	-.01713*	0.00349	0.000	-0.0265	-0.0078
		Class 3 obesity	-.01306*	0.00335	0.001	-0.0220	-0.0041
		Class 4 obesity	-.02350*	0.00315	0.000	-0.0319	-0.0151
	Class 3 obesity	Normal body fat	-0.00407	0.00331	1.000	-0.0130	0.0048
		Class 1 and 2 obesity	.01306*	0.00335	0.001	0.0041	0.0220
		Class 4 obesity	-.01044*	0.00295	0.003	-0.0184	-0.0025
	Class 4 obesity	Normal body fat	0.00637	0.00311	0.256	-0.0020	0.0147
		Class 1 and 2 obesity	.02350*	0.00315	0.000	0.0151	0.0319
		Class 3 obesity	.01044*	0.00295	0.003	0.0025	0.0184
SBP (mmHg)	Normal body fat	Class 1 and 2 obesity	-0.05581	0.02907	0.343	-0.1338	0.0222
		Class 3 obesity	-.10385*	0.02759	0.002	-0.1779	-0.0298
		Class 4 obesity	-.14093*	0.02589	0.000	-0.2104	-0.0715
	Class 1 and 2 obesity	Normal body fat	0.05581	0.02907	0.343	-0.0222	0.1338
		Class 3 obesity	-0.04805	0.02789	0.525	-0.1229	0.0268
		Class 4 obesity	-.08513*	0.02621	0.009	-0.1554	-0.0148
	Class 3 obesity	Normal body fat	.10385*	0.02759	0.002	0.0298	0.1779
		Class 1 and 2 obesity	0.04805	0.02789	0.525	-0.0268	0.1229
		Class 4 obesity	-0.03708	0.02457	0.803	-0.1030	0.0288
	Class 4 obesity	Normal body fat	.14093*	0.02589	0.000	0.0715	0.2104
		Class 1 and 2 obesity	.08513*	0.02621	0.009	0.0148	0.1554
		Class 3 obesity	0.03708	0.02457	0.803	-0.0288	0.1030
DBP (mmHg)	Normal body fat	Class 1 and 2 obesity	-0.23400	0.15887	0.860	-0.6602	0.1922
		Class 3 obesity	-.47130*	0.15083	0.013	-0.8759	-0.0667
		Class 4 obesity	-0.34448	0.14153	0.098	-0.7242	0.0352
	Class 1 and 2 obesity	Normal body fat	0.23400	0.15887	0.860	-0.1922	0.6602
		Class 3 obesity	-0.23730	0.15246	0.733	-0.6463	0.1717
		Class 4 obesity	-0.11048	0.14327	1.000	-0.4948	0.2739
	Class 3 obesity	Normal body fat	.47130*	0.15083	0.013	0.0667	0.8759
		Class 1 and 2 obesity	0.23730	0.15246	0.733	-0.1717	0.6463
		Class 4 obesity	0.12682	0.13430	1.000	-0.2335	0.4871
	Class 4 obesity	Normal body fat	0.34448	0.14153	0.098	-0.0352	0.7242
		Class 1 and 2 obesity	0.11048	0.14327	1.000	-0.2739	0.4948
		Class 3 obesity	-0.12682	0.13430	1.000	-0.4871	0.2335

*. The difference in means is significant at the 0.05 level.

Table 15S. **Bonferroni test post hoc. Immune cells relative values**							
**ANOVA, multiple comparisons**							
			difference of means (I-J)	Desv. Error	Sig.	Confidence interval 95%	
						Lower limit	Upper limit
Total lymphocytes (%)	Normal body fat	Class 1 and 2 obesity	-7.87585	3.38240	0.130	-16.9524	1.2007
		Class 3 obesity	-4.52233	3.14230	0.916	-12.9546	3.9099
		Class 4 obesity	-2.06441	2.94863	1.000	-9.9770	5.8481
	Class 1 and 2 obesity	Normal body fat	7.87585	3.38240	0.130	-1.2007	16.9524
		Class 3 obesity	3.35352	3.25185	1.000	-5.3727	12.0797
		Class 4 obesity	5.81144	3.06511	0.362	-2.4137	14.0365
	Class 3 obesity	Normal body fat	4.52233	3.14230	0.916	-3.9099	12.9546
		Class 1 and 2 obesity	-3.35352	3.25185	1.000	-12.0797	5.3727
		Class 4 obesity	2.45792	2.79791	1.000	-5.0502	9.9660
	Class 4 obesity	Normal body fat	2.06441	2.94863	1.000	-5.8481	9.9770
		Class 1 and 2 obesity	-5.81144	3.06511	0.362	-14.0365	2.4137
		Class 3 obesity	-2.45792	2.79791	1.000	-9.9660	5.0502
Monocytes (%)	Normal body fat	Class 1 and 2 obesity	-0.45967	0.80921	1.000	-2.6312	1.7118
		Class 3 obesity	-0.01278	0.75177	1.000	-2.0301	2.0046
		Class 4 obesity	-0.30185	0.70544	1.000	-2.1949	1.5912
	Class 1 and 2 obesity	Normal body fat	0.45967	0.80921	1.000	-1.7118	2.6312
		Class 3 obesity	0.44689	0.77798	1.000	-1.6408	2.5346
		Class 4 obesity	0.15782	0.73330	1.000	-1.8100	2.1256
	Class 3 obesity	Normal body fat	0.01278	0.75177	1.000	-2.0046	2.0301
		Class 1 and 2 obesity	-0.44689	0.77798	1.000	-2.5346	1.6408
		Class 4 obesity	-0.28907	0.66938	1.000	-2.0853	1.5072
	Class 4 obesity	Normal body fat	0.30185	0.70544	1.000	-1.5912	2.1949
		Class 1 and 2 obesity	-0.15782	0.73330	1.000	-2.1256	1.8100
		Class 3 obesity	0.28907	0.66938	1.000	-1.5072	2.0853
Granulocytes (%)	Normal body fat	Class 1 and 2 obesity	8.31821	3.44253	0.103	-0.9197	17.5561
		Class 3 obesity	4.20361	3.19816	1.000	-4.3785	12.7858
		Class 4 obesity	1.99396	3.00105	1.000	-6.0593	10.0472
	Class 1 and 2 obesity	Normal body fat	-8.31821	3.44253	0.103	-17.5561	0.9197
		Class 3 obesity	-4.11460	3.30965	1.000	-12.9959	4.7667
		Class 4 obesity	-6.32425	3.11960	0.269	-14.6956	2.0471
	Class 3 obesity	Normal body fat	-4.20361	3.19816	1.000	-12.7858	4.3785
		Class 1 and 2 obesity	4.11460	3.30965	1.000	-4.7667	12.9959
		Class 4 obesity	-2.20965	2.84765	1.000	-9.8512	5.4319
	Class 4 obesity	Normal body fat	-1.99396	3.00105	1.000	-10.0472	6.0593
		Class 1 and 2 obesity	6.32425	3.11960	0.269	-2.0471	14.6956
		Class 3 obesity	2.20965	2.84765	1.000	-5.4319	9.8512
TCD4+ lymphocytes (%)	Normal body fat	Class 1 and 2 obesity	-3.34676	3.19485	1.000	-11.9200	5.2265
		Class 3 obesity	-9.24702*	2.96807	0.014	-17.2117	-1.2823
		Class 4 obesity	-10.25440*	2.78514	0.002	-17.7282	-2.7806
	Class 1 and 2 obesity	Normal body fat	3.34676	3.19485	1.000	-5.2265	11.9200
		Class 3 obesity	-5.90027	3.07154	0.343	-14.1426	2.3421
		Class 4 obesity	-6.90764	2.89515	0.112	-14.6767	0.8614
	Class 3 obesity	Normal body fat	9.24702*	2.96807	0.014	1.2823	17.2117
		Class 1 and 2 obesity	5.90027	3.07154	0.343	-2.3421	14.1426
		Class 4 obesity	-1.00737	2.64277	1.000	-8.0992	6.0844
		Normal body fat	10.25440*	2.78514	0.002	2.7806	17.7282

	Class 4 obesity	Class 1 and 2 obesity	6.90764	2.89515	0.112	-0.8614	14.6767
		Class 3 obesity	1.00737	2.64277	1.000	-6.0844	8.0992
TCD4+CD62+ lymphocytes (%)	Normal body fat	Class 1 and 2 obesity	16.27805*	5.87590	0.039	0.4906	32.0655
		Class 3 obesity	21.39164*	5.08800	0.000	7.7212	35.0621
		Class 4 obesity	20.96923*	4.70105	0.000	8.3384	33.6000
	Class 1 and 2 obesity	Normal body fat	-16.27805*	5.87590	0.039	-32.0655	-0.4906
		Class 3 obesity	5.11359	5.75449	1.000	-10.3476	20.5748
		Class 4 obesity	4.69118	5.41537	1.000	-9.8589	19.2412
	Class 3 obesity	Normal body fat	-21.39164*	5.08800	0.000	-35.0621	-7.7212
		Class 1 and 2 obesity	-5.11359	5.75449	1.000	-20.5748	10.3476
		Class 4 obesity	-0.42241	4.54838	1.000	-12.6430	11.7982
	Class 4 obesity	Normal body fat	-20.96923*	4.70105	0.000	-33.6000	-8.3384
		Class 1 and 2 obesity	-4.69118	5.41537	1.000	-19.2412	9.8589
		Class 3 obesity	0.42241	4.54838	1.000	-11.7982	12.6430
TCD4+CD62- lymphocytes (%)	Normal body fat	Class 1 and 2 obesity	-12.35498	5.70141	0.194	-27.6736	2.9636
		Class 3 obesity	-13.62374*	4.93690	0.041	-26.8883	-0.3592
		Class 4 obesity	-20.49139*	4.56144	0.000	-32.7471	-8.2357
	Class 1 and 2 obesity	Normal body fat	12.35498	5.70141	0.194	-2.9636	27.6736
		Class 3 obesity	-1.26876	5.58360	1.000	-16.2708	13.7333
		Class 4 obesity	-8.13641	5.25455	0.746	-22.2544	5.9816
	Class 3 obesity	Normal body fat	13.62374*	4.93690	0.041	0.3592	26.8883
		Class 1 and 2 obesity	1.26876	5.58360	1.000	-13.7333	16.2708
		Class 4 obesity	-6.86765	4.41331	0.735	-18.7254	4.9901
	Class 4 obesity	Normal body fat	20.49139*	4.56144	0.000	8.2357	32.7471
		Class 1 and 2 obesity	8.13641	5.25455	0.746	-5.9816	22.2544
		Class 3 obesity	6.86765	4.41331	0.735	-4.9901	18.7254
TCD8+CD28+ lymphocytes (%)	Normal body fat	Class 1 and 2 obesity	-11.27207	5.49039	0.255	-26.0366	3.4925
		Class 3 obesity	-3.51827	5.42936	1.000	-18.1187	11.0822
		Class 4 obesity	-7.42521	4.93644	0.813	-20.7001	5.8497
	Class 1 and 2 obesity	Normal body fat	11.27207	5.49039	0.255	-3.4925	26.0366
		Class 3 obesity	7.75380	5.59674	1.000	-7.2967	22.8043
		Class 4 obesity	3.84686	5.11996	1.000	-9.9215	17.6153
	Class 3 obesity	Normal body fat	3.51827	5.42936	1.000	-11.0822	18.1187
		Class 1 and 2 obesity	-7.75380	5.59674	1.000	-22.8043	7.2967
		Class 4 obesity	-3.90694	5.05446	1.000	-17.4992	9.6853
	Class 4 obesity	Normal body fat	7.42521	4.93644	0.813	-5.8497	20.7001
		Class 1 and 2 obesity	-3.84686	5.11996	1.000	-17.6153	9.9215
		Class 3 obesity	3.90694	5.05446	1.000	-9.6853	17.4992
TCD8+CD28- lymphocytes (%)	Normal body fat	Class 1 and 2 obesity	7.32937	5.80888	1.000	-8.2780	22.9367
		Class 3 obesity	2.03146	5.37299	1.000	-12.4047	16.4677
		Class 4 obesity	5.21902	5.16092	1.000	-8.6474	19.0854
	Class 1 and 2 obesity	Normal body fat	-7.32937	5.80888	1.000	-22.9367	8.2780
		Class 3 obesity	-5.29791	5.62858	1.000	-20.4208	9.8250
		Class 4 obesity	-2.11035	5.42651	1.000	-16.6904	12.4696
	Class 3 obesity	Normal body fat	-2.03146	5.37299	1.000	-16.4677	12.4047
		Class 1 and 2 obesity	5.29791	5.62858	1.000	-9.8250	20.4208
		Class 4 obesity	3.18756	4.95712	1.000	-10.1313	16.5064
	Class 4 obesity	Normal body fat	-5.21902	5.16092	1.000	-19.0854	8.6474
		Class 1 and 2 obesity	2.11035	5.42651	1.000	-12.4696	16.6904
		Class 3 obesity	-3.18756	4.95712	1.000	-16.5064	10.1313

TCD8+CD45RA+ lymphocytes (%)	Normal body fat	Class 1 and 2 obesity	-0.86667	5.24519	1.000	-15.0173	13.2839
		Class 3 obesity	10.00505	5.00109	0.291	-3.4870	23.4971
		Class 4 obesity	9.83611	4.54246	0.198	-2.4187	22.0909
	Class 1 and 2 obesity	Normal body fat	0.86667	5.24519	1.000	-13.2839	15.0173
		Class 3 obesity	10.87172	5.00109	0.194	-2.6203	24.3638
		Class 4 obesity	10.70278	4.54246	0.124	-1.5520	22.9576
	Class 3 obesity	Normal body fat	-10.00505	5.00109	0.291	-23.4971	3.4870
		Class 1 and 2 obesity	-10.87172	5.00109	0.194	-24.3638	2.6203
		Class 4 obesity	-0.16894	4.25827	1.000	-11.6570	11.3191
	Class 4 obesity	Normal body fat	-9.83611	4.54246	0.198	-22.0909	2.4187
		Class 1 and 2 obesity	-10.70278	4.54246	0.124	-22.9576	1.5520
		Class 3 obesity	0.16894	4.25827	1.000	-11.3191	11.6570
TCD8+CD45RO+ lymphocytes (%)	Normal body fat	Class 1 and 2 obesity	0.67222	6.05175	1.000	-15.6350	16.9794
		Class 3 obesity	-7.81111	5.56680	0.983	-22.8116	7.1893
		Class 4 obesity	-13.74625	5.21730	0.059	-27.8049	0.3124
	Class 1 and 2 obesity	Normal body fat	-0.67222	6.05175	1.000	-16.9794	15.6350
		Class 3 obesity	-8.48333	5.56680	0.785	-23.4838	6.5171
		Class 4 obesity	-14.41847*	5.21730	0.041	-28.4772	-0.3598
	Class 3 obesity	Normal body fat	7.81111	5.56680	0.983	-7.1893	22.8116
		Class 1 and 2 obesity	8.48333	5.56680	0.785	-6.5171	23.4838
		Class 4 obesity	-5.93514	4.64606	1.000	-18.4545	6.5843
	Class 4 obesity	Normal body fat	13.74625	5.21730	0.059	-0.3124	27.8049
		Class 1 and 2 obesity	14.41847*	5.21730	0.041	0.3598	28.4772
		Class 3 obesity	5.93514	4.64606	1.000	-6.5843	18.4545
NK lymphocytes (%)	Normal body fat	Class 1 and 2 obesity	0.31464	0.16459	0.351	-0.1273	0.7566
		Class 3 obesity	-0.01301	0.15151	1.000	-0.4198	0.3938
		Class 4 obesity	0.01702	0.13999	1.000	-0.3589	0.3929
	Class 1 and 2 obesity	Normal body fat	-0.31464	0.16459	0.351	-0.7566	0.1273
		Class 3 obesity	-0.32765	0.16074	0.263	-0.7592	0.1039
		Class 4 obesity	-0.29762	0.14993	0.297	-0.7002	0.1049
	Class 3 obesity	Normal body fat	0.01301	0.15151	1.000	-0.3938	0.4198
		Class 1 and 2 obesity	0.32765	0.16074	0.263	-0.1039	0.7592
		Class 4 obesity	0.03004	0.13544	1.000	-0.3336	0.3937
	Class 4 obesity	Normal body fat	-0.01702	0.13999	1.000	-0.3929	0.3589
		Class 1 and 2 obesity	0.29762	0.14993	0.297	-0.1049	0.7002
		Class 3 obesity	-0.03004	0.13544	1.000	-0.3937	0.3336
NKT lymphocytes (%)	Normal body fat	Class 1 and 2 obesity	-0.09228	0.54816	1.000	-1.5848	1.4002
		Class 3 obesity	0.09878	0.36761	1.000	-0.9021	1.0997
		Class 4 obesity	0.05729	0.33319	1.000	-0.8499	0.9645
	Class 1 and 2 obesity	Normal body fat	0.09228	0.54816	1.000	-1.4002	1.5848
		Class 3 obesity	0.19106	0.54086	1.000	-1.2816	1.6637
		Class 4 obesity	0.14957	0.51808	1.000	-1.2610	1.5602
	Class 3 obesity	Normal body fat	-0.09878	0.36761	1.000	-1.0997	0.9021
		Class 1 and 2 obesity	-0.19106	0.54086	1.000	-1.6637	1.2816
		Class 4 obesity	-0.04149	0.32104	1.000	-0.9156	0.8326
	Class 4 obesity	Normal body fat	-0.05729	0.33319	1.000	-0.9645	0.8499
		Class 1 and 2 obesity	-0.14957	0.51808	1.000	-1.5602	1.2610
		Class 3 obesity	0.04149	0.32104	1.000	-0.8326	0.9156
TCD8+ lymphocytes (%)		Class 1 and 2 obesity	-0.02402	0.00985	0.098	-0.0505	0.0024

	Normal body fat	Class 3 obesity	-0.02316	0.00929	0.085	-0.0481	0.0018
		Class 4 obesity	-.03760*	0.00871	0.000	-0.0610	-0.0142
	Class 1 and 2 obesity	Normal body fat	0.02402	0.00985	0.098	-0.0024	0.0505
		Class 3 obesity	0.00086	0.00961	1.000	-0.0249	0.0267
		Class 4 obesity	-0.01358	0.00905	0.816	-0.0379	0.0107
	Class 3 obesity	Normal body fat	0.02316	0.00929	0.085	-0.0018	0.0481
		Class 1 and 2 obesity	-0.00086	0.00961	1.000	-0.0267	0.0249
		Class 4 obesity	-0.01444	0.00844	0.539	-0.0371	0.0082
	Class 4 obesity	Normal body fat	.03760*	0.00871	0.000	0.0142	0.0610
		Class 1 and 2 obesity	0.01358	0.00905	0.816	-0.0107	0.0379
		Class 3 obesity	0.01444	0.00844	0.539	-0.0082	0.0371
TCD4+CD45RA+ lymphocytes (%)	Normal body fat	Class 1 and 2 obesity	-0.41148	0.20145	0.264	-0.9547	0.1317
		Class 3 obesity	-0.02642	0.19208	1.000	-0.5444	0.4915
		Class 4 obesity	-0.16185	0.17293	1.000	-0.6281	0.3044
	Class 1 and 2 obesity	Normal body fat	0.41148	0.20145	0.264	-0.1317	0.9547
		Class 3 obesity	0.38506	0.19208	0.288	-0.1329	0.9030
		Class 4 obesity	0.24963	0.17293	0.914	-0.2167	0.7159
	Class 3 obesity	Normal body fat	0.02642	0.19208	1.000	-0.4915	0.5444
		Class 1 and 2 obesity	-0.38506	0.19208	0.288	-0.9030	0.1329
		Class 4 obesity	-0.13543	0.16191	1.000	-0.5720	0.3012
	Class 4 obesity	Normal body fat	0.16185	0.17293	1.000	-0.3044	0.6281
		Class 1 and 2 obesity	-0.24963	0.17293	0.914	-0.7159	0.2167
		Class 3 obesity	0.13543	0.16191	1.000	-0.3012	0.5720
TCD4+CD45RA+CD45RO+ lymphocytes (%)	Normal body fat	Class 1 and 2 obesity	-0.22153	0.15638	0.959	-0.6430	0.1999
		Class 3 obesity	-0.15115	0.14628	1.000	-0.5454	0.2431
		Class 4 obesity	-0.16389	0.13424	1.000	-0.5257	0.1979
	Class 1 and 2 obesity	Normal body fat	0.22153	0.15638	0.959	-0.1999	0.6430
		Class 3 obesity	0.07038	0.14628	1.000	-0.3239	0.4646
		Class 4 obesity	0.05765	0.13424	1.000	-0.3041	0.4194
	Class 3 obesity	Normal body fat	0.15115	0.14628	1.000	-0.2431	0.5454
		Class 1 and 2 obesity	-0.07038	0.14628	1.000	-0.4646	0.3239
		Class 4 obesity	-0.01273	0.12232	1.000	-0.3424	0.3169
	Class 4 obesity	Normal body fat	0.16389	0.13424	1.000	-0.1979	0.5257
		Class 1 and 2 obesity	-0.05765	0.13424	1.000	-0.4194	0.3041
		Class 3 obesity	0.01273	0.12232	1.000	-0.3169	0.3424
TCD4+CD45RO+ lymphocytes (%)	Normal body fat	Class 1 and 2 obesity	1629.56667	822.35942	0.302	-585.8929	3845.0262
		Class 3 obesity	-109.32231	756.46064	1.000	-2147.2487	1928.6040
		Class 4 obesity	402.72447	705.90928	1.000	-1499.0151	2304.4640
	Class 1 and 2 obesity	Normal body fat	-1629.5666	822.35942	0.302	-3845.0262	585.8929
		Class 3 obesity	-1738.8889	756.46064	0.142	-3776.8153	299.0374
		Class 4 obesity	-1226.8421	705.90928	0.513	-3128.5817	674.8973
	Class 3 obesity	Normal body fat	109.32231	756.46064	1.000	-1928.6040	2147.2487
		Class 1 and 2 obesity	1738.88897	756.46064	0.142	-299.0374	3776.8153
		Class 4 obesity	512.04678	627.90572	1.000	-1179.5490	2203.6425
	Class 4 obesity	Normal body fat	-402.72447	705.90928	1.000	-2304.4640	1499.0151
		Class 1 and 2 obesity	1226.84219	705.90928	0.513	-674.8973	3128.5817
		Class 3 obesity	-512.04678	627.90572	1.000	-2203.6425	1179.5490
TCD8+CD45RA+CD45RO+ lymphocytes (%)	Normal body fat	Class 1 and 2 obesity	0.14472	0.17433	1.000	-0.3251	0.6146
		Class 3 obesity	0.06853	0.16078	1.000	-0.3648	0.5019
		Class 4 obesity	0.11346	0.15103	1.000	-0.2936	0.5205

	Class 1 and 2 obesity	Normal body fat	-0.14472	0.17433	1.000	-0.6146	0.3251
		Class 3 obesity	-0.07619	0.15805	1.000	-0.5022	0.3498
		Class 4 obesity	-0.03127	0.14813	1.000	-0.4305	0.3680
	Class 3 obesity	Normal body fat	-0.06853	0.16078	1.000	-0.5019	0.3648
		Class 1 and 2 obesity	0.07619	0.15805	1.000	-0.3498	0.5022
		Class 4 obesity	0.04493	0.13191	1.000	-0.3106	0.4005
	Class 4 obesity	Normal body fat	-0.11346	0.15103	1.000	-0.5205	0.2936
		Class 1 and 2 obesity	0.03127	0.14813	1.000	-0.3680	0.4305
		Class 3 obesity	-0.04493	0.13191	1.000	-0.4005	0.3106
TCD3+ lymphocytes (%)	Normal body fat	Class 1 and 2 obesity	-0.20717	0.38503	1.000	-1.2401	0.8257
		Class 3 obesity	-0.66830	0.36554	0.420	-1.6489	0.3123
		Class 4 obesity	-0.70151	0.34301	0.258	-1.6217	0.2187
	Class 1 and 2 obesity	Normal body fat	0.20717	0.38503	1.000	-0.8257	1.2401
		Class 3 obesity	-0.46112	0.36949	1.000	-1.4524	0.5301
		Class 4 obesity	-0.49433	0.34722	0.943	-1.4258	0.4372
	Class 3 obesity	Normal body fat	0.66830	0.36554	0.420	-0.3123	1.6489
		Class 1 and 2 obesity	0.46112	0.36949	1.000	-0.5301	1.4524
		Class 4 obesity	-0.03321	0.32548	1.000	-0.9064	0.8399
	Class 4 obesity	Normal body fat	0.70151	0.34301	0.258	-0.2187	1.6217
		Class 1 and 2 obesity	0.49433	0.34722	0.943	-0.4372	1.4258
		Class 3 obesity	0.03321	0.32548	1.000	-0.8399	0.9064
B lymphocytes (%)	Normal body fat	Class 1 and 2 obesity	0.18618	0.09589	0.327	-0.0711	0.4434
		Class 3 obesity	0.20845	0.09104	0.143	-0.0358	0.4527
		Class 4 obesity	.25580*	0.08543	0.020	0.0266	0.4850
	Class 1 and 2 obesity	Normal body fat	-0.18618	0.09589	0.327	-0.4434	0.0711
		Class 3 obesity	0.02227	0.09202	1.000	-0.2246	0.2691
		Class 4 obesity	0.06963	0.08648	1.000	-0.1624	0.3016
	Class 3 obesity	Normal body fat	-0.20845	0.09104	0.143	-0.4527	0.0358
		Class 1 and 2 obesity	-0.02227	0.09202	1.000	-0.2691	0.2246
		Class 4 obesity	0.04736	0.08106	1.000	-0.1701	0.2648
	Class 4 obesity	Normal body fat	-.25580*	0.08543	0.020	-0.4850	-0.0266
		Class 1 and 2 obesity	-0.06963	0.08648	1.000	-0.3016	0.1624
		Class 3 obesity	-0.04736	0.08106	1.000	-0.2648	0.1701

*. The difference in means is significant at the 0.05 level.

Table 16S. **Bonferroni test post hoc. Immune cells absolute values**

ANOVA, multiple comparisons

			difference of means (I-J)	Desv. Error	Sig.	Confidence interval 95%	
						Lower limit	Upper limit
Leukocytes (cel/µL)	Normal body fat	Class 1 and 2 obesity	-0.17189	0.17737	1.000	-0.6516	0.3078
		Class 3 obesity	-.39584*	0.12086	0.009	-0.7227	-0.0690
		Class 4 obesity	-0.27259	0.10679	0.075	-0.5614	0.0162
	Class 1 and 2 obesity	Normal body fat	0.17189	0.17737	1.000	-0.3078	0.6516
		Class 3 obesity	-0.22395	0.17417	1.000	-0.6950	0.2471
		Class 4 obesity	-0.10070	0.16472	1.000	-0.5462	0.3448
		Normal body fat	.39584*	0.12086	0.009	0.0690	0.7227

	Class 3 obesity	Class 1 and 2 obesity	0.22395	0.17417	1.000	-0.2471	0.6950
		Class 4 obesity	0.12325	0.10139	1.000	-0.1510	0.3975
	Class 4 obesity	Normal body fat	0.27259	0.10679	0.075	-0.0162	0.5614
		Class 1 and 2 obesity	0.10070	0.16472	1.000	-0.3448	0.5462
		Class 3 obesity	-0.12325	0.10139	1.000	-0.3975	0.1510
Total lymphocytes (cel/μL)	Normal body fat	Class 1 and 2 obesity	-0.14725	0.23833	1.000	-0.7917	0.4971
		Class 3 obesity	-.72277*	0.16240	0.000	-1.1619	-0.2837
		Class 4 obesity	-.55804*	0.14295	0.001	-0.9446	-0.1715
	Class 1 and 2 obesity	Normal body fat	0.14725	0.23833	1.000	-0.4971	0.7917
		Class 3 obesity	-0.57552	0.23404	0.096	-1.2083	0.0573
		Class 4 obesity	-0.41079	0.22099	0.400	-1.0083	0.1867
	Class 3 obesity	Normal body fat	.72277*	0.16240	0.000	0.2837	1.1619
		Class 1 and 2 obesity	0.57552	0.23404	0.096	-0.0573	1.2083
		Class 4 obesity	0.16473	0.13567	1.000	-0.2021	0.5316
	Class 4 obesity	Normal body fat	.55804*	0.14295	0.001	0.1715	0.9446
		Class 1 and 2 obesity	0.41079	0.22099	0.400	-0.1867	1.0083
		Class 3 obesity	-0.16473	0.13567	1.000	-0.5316	0.2021
Monocytes (cel/μL)	Normal body fat	Class 1 and 2 obesity	0.00406	0.00595	1.000	-0.0120	0.0202
		Class 3 obesity	0.00762	0.00405	0.382	-0.0033	0.0186
		Class 4 obesity	0.00807	0.00357	0.158	-0.0016	0.0177
	Class 1 and 2 obesity	Normal body fat	-0.00406	0.00595	1.000	-0.0202	0.0120
		Class 3 obesity	0.00355	0.00584	1.000	-0.0122	0.0194
		Class 4 obesity	0.00401	0.00552	1.000	-0.0109	0.0189
	Class 3 obesity	Normal body fat	-0.00762	0.00405	0.382	-0.0186	0.0033
		Class 1 and 2 obesity	-0.00355	0.00584	1.000	-0.0194	0.0122
		Class 4 obesity	0.00045	0.00339	1.000	-0.0087	0.0096
	Class 4 obesity	Normal body fat	-0.00807	0.00357	0.158	-0.0177	0.0016
		Class 1 and 2 obesity	-0.00401	0.00552	1.000	-0.0189	0.0109
		Class 3 obesity	-0.00045	0.00339	1.000	-0.0096	0.0087
Granulocytes (cel/μL)	Normal body fat	Class 1 and 2 obesity	-0.16922	0.19376	1.000	-0.6931	0.3547
		Class 3 obesity	-0.26558	0.13202	0.285	-0.6225	0.0914
		Class 4 obesity	-0.17054	0.11621	0.876	-0.4848	0.1437
	Class 1 and 2 obesity	Normal body fat	0.16922	0.19376	1.000	-0.3547	0.6931
		Class 3 obesity	-0.09636	0.19027	1.000	-0.6108	0.4181
		Class 4 obesity	-0.00132	0.17966	1.000	-0.4871	0.4844
	Class 3 obesity	Normal body fat	0.26558	0.13202	0.285	-0.0914	0.6225
		Class 1 and 2 obesity	0.09636	0.19027	1.000	-0.4181	0.6108
		Class 4 obesity	0.09504	0.11030	1.000	-0.2032	0.3933
	Class 4 obesity	Normal body fat	0.17054	0.11621	0.876	-0.1437	0.4848
		Class 1 and 2 obesity	0.00132	0.17966	1.000	-0.4844	0.4871
		Class 3 obesity	-0.09504	0.11030	1.000	-0.3933	0.2032
TCD3+ lymphocytes (cel/μL)	Normal body fat	Class 1 and 2 obesity	-0.28845	0.27593	1.000	-1.0347	0.4578
		Class 3 obesity	-.80586*	0.18958	0.000	-1.3186	-0.2931
		Class 4 obesity	-.73160*	0.16763	0.000	-1.1850	-0.2782

	Class 1 and 2 obesity	Normal body fat	0.28845	0.27593	1.000	-0.4578	1.0347
		Class 3 obesity	-0.51741	0.26899	0.348	-1.2449	0.2101
		Class 4 obesity	-0.44315	0.25399	0.509	-1.1301	0.2438
	Class 3 obesity	Normal body fat	.80586*	0.18958	0.000	0.2931	1.3186
		Class 1 and 2 obesity	0.51741	0.26899	0.348	-0.2101	1.2449
		Class 4 obesity	0.07426	0.15593	1.000	-0.3475	0.4960
	Class 4 obesity	Normal body fat	.73160*	0.16763	0.000	0.2782	1.1850
		Class 1 and 2 obesity	0.44315	0.25399	0.509	-0.2438	1.1301
		Class 3 obesity	-0.07426	0.15593	1.000	-0.4960	0.3475
NK lymphocytes (cel/µL)	Normal body fat	Class 1 and 2 obesity	0.27049	0.30818	1.000	-0.5633	1.1043
		Class 3 obesity	-0.50373	0.21503	0.130	-1.0855	0.0780
		Class 4 obesity	-0.13612	0.18485	1.000	-0.6362	0.3640
	Class 1 and 2 obesity	Normal body fat	-0.27049	0.30818	1.000	-1.1043	0.5633
		Class 3 obesity	-0.77422	0.30615	0.080	-1.6025	0.0540
		Class 4 obesity	-0.40661	0.28576	0.952	-1.1797	0.3665
	Class 3 obesity	Normal body fat	0.50373	0.21503	0.130	-0.0780	1.0855
		Class 1 and 2 obesity	0.77422	0.30615	0.080	-0.0540	1.6025
		Class 4 obesity	0.36762	0.18144	0.276	-0.1233	0.8585
	Class 4 obesity	Normal body fat	0.13612	0.18485	1.000	-0.3640	0.6362
		Class 1 and 2 obesity	0.40661	0.28576	0.952	-0.3665	1.1797
		Class 3 obesity	-0.36762	0.18144	0.276	-0.8585	0.1233
NKT lymphocytes (cel/µL)	Normal body fat	Class 1 and 2 obesity	0.02810	0.53439	1.000	-1.4276	1.4838
		Class 3 obesity	-0.32287	0.36178	1.000	-1.3084	0.6627
		Class 4 obesity	-0.15992	0.32912	1.000	-1.0565	0.7366
	Class 1 and 2 obesity	Normal body fat	-0.02810	0.53439	1.000	-1.4838	1.4276
		Class 3 obesity	-0.35097	0.52314	1.000	-1.7761	1.0741
		Class 4 obesity	-0.18802	0.50110	1.000	-1.5531	1.1770
	Class 3 obesity	Normal body fat	0.32287	0.36178	1.000	-0.6627	1.3084
		Class 1 and 2 obesity	0.35097	0.52314	1.000	-1.0741	1.7761
		Class 4 obesity	0.16295	0.31052	1.000	-0.6829	1.0088
	Class 4 obesity	Normal body fat	0.15992	0.32912	1.000	-0.7366	1.0565
		Class 1 and 2 obesity	0.18802	0.50110	1.000	-1.1770	1.5531
		Class 3 obesity	-0.16295	0.31052	1.000	-1.0088	0.6829
B lymphocytes (cel/µL)	Normal body fat	Class 1 and 2 obesity	-0.23695	0.33134	1.000	-1.1336	0.6597
		Class 3 obesity	-0.39724	0.21294	0.395	-0.9735	0.1791
		Class 4 obesity	-0.26960	0.18603	0.907	-0.7730	0.2338
	Class 1 and 2 obesity	Normal body fat	0.23695	0.33134	1.000	-0.6597	1.1336
		Class 3 obesity	-0.16029	0.32771	1.000	-1.0472	0.7266
		Class 4 obesity	-0.03265	0.31090	1.000	-0.8740	0.8087
	Class 3 obesity	Normal body fat	0.39724	0.21294	0.395	-0.1791	0.9735
		Class 1 and 2 obesity	0.16029	0.32771	1.000	-0.7266	1.0472
		Class 4 obesity	0.12764	0.17950	1.000	-0.3581	0.6134

	Class 4 obesity	Normal body fat	0.26960	0.18603	0.907	-0.2338	0.7730
		Class 1 and 2 obesity	0.03265	0.31090	1.000	-0.8087	0.8740
		Class 3 obesity	-0.12764	0.17950	1.000	-0.6134	0.3581
TCD4+ lymphocytes (cel/μL) (para cd62)	Normal body fat	Class 1 and 2 obesity	-0.32581	0.24047	1.000	-0.9760	0.3244
		Class 3 obesity	-.84717*	0.16385	0.000	-1.2902	-0.4042
		Class 4 obesity	-.79544*	0.14423	0.000	-1.1854	-0.4055
	Class 1 and 2 obesity	Normal body fat	0.32581	0.24047	1.000	-0.3244	0.9760
		Class 3 obesity	-0.52136	0.23614	0.180	-1.1598	0.1171
		Class 4 obesity	-0.46962	0.22297	0.229	-1.0725	0.1332
	Class 3 obesity	Normal body fat	.84717*	0.16385	0.000	0.4042	1.2902
		Class 1 and 2 obesity	0.52136	0.23614	0.180	-0.1171	1.1598
		Class 4 obesity	0.05174	0.13689	1.000	-0.3184	0.4218
	Class 4 obesity	Normal body fat	.79544*	0.14423	0.000	0.4055	1.1854
		Class 1 and 2 obesity	0.46962	0.22297	0.229	-0.1332	1.0725
		Class 3 obesity	-0.05174	0.13689	1.000	-0.4218	0.3184
TCD4+CD62+ lymphocytes (cel/μL)	Normal body fat	Class 1 and 2 obesity	2.22506	5.38931	1.000	-12.3464	16.7965
		Class 3 obesity	-6.02866	3.67219	0.627	-15.9574	3.9001
		Class 4 obesity	-3.66756	3.23249	1.000	-12.4075	5.0724
	Class 1 and 2 obesity	Normal body fat	-2.22506	5.38931	1.000	-16.7965	12.3464
		Class 3 obesity	-8.25372	5.29220	0.736	-22.5626	6.0552
		Class 4 obesity	-5.89262	4.99713	1.000	-19.4037	7.6185
	Class 3 obesity	Normal body fat	6.02866	3.67219	0.627	-3.9001	15.9574
		Class 1 and 2 obesity	8.25372	5.29220	0.736	-6.0552	22.5626
		Class 4 obesity	2.36110	3.06785	1.000	-5.9336	10.6559
	Class 4 obesity	Normal body fat	3.66756	3.23249	1.000	-5.0724	12.4075
		Class 1 and 2 obesity	5.89262	4.99713	1.000	-7.6185	19.4037
		Class 3 obesity	-2.36110	3.06785	1.000	-10.6559	5.9336
TCD4+CD62- lymphocytes (cel/μL)	Normal body fat	Class 1 and 2 obesity	-7.37035	4.34202	0.560	-19.1102	4.3695
		Class 3 obesity	-16.98818*	2.95859	0.000	-24.9875	-8.9888
		Class 4 obesity	-15.26101*	2.60433	0.000	-22.3025	-8.2195
	Class 1 and 2 obesity	Normal body fat	7.37035	4.34202	0.560	-4.3695	19.1102
		Class 3 obesity	-9.61783	4.26378	0.161	-21.1461	1.9105
		Class 4 obesity	-7.89066	4.02606	0.320	-18.7762	2.9949
	Class 3 obesity	Normal body fat	16.98818*	2.95859	0.000	8.9888	24.9875
		Class 1 and 2 obesity	9.61783	4.26378	0.161	-1.9105	21.1461
		Class 4 obesity	1.72717	2.47168	1.000	-4.9557	8.4100
	Class 4 obesity	Normal body fat	15.26101*	2.60433	0.000	8.2195	22.3025
		Class 1 and 2 obesity	7.89066	4.02606	0.320	-2.9949	18.7762
		Class 3 obesity	-1.72717	2.47168	1.000	-8.4100	4.9557
TCD8+ lymphocytes (cel/μL)	Normal body fat	Class 1 and 2 obesity	-0.01038	0.00546	0.367	-0.0252	0.0044
		Class 3 obesity	0.00436	0.00381	1.000	-0.0060	0.0147

		Class 4 obesity	-0.00211	0.00332	1.000	-0.0111	0.0069
	Class 1 and 2 obesity	Normal body fat	0.01038	0.00546	0.367	-0.0044	0.0252
		Class 3 obesity	.01474*	0.00543	0.049	0.0000	0.0294
		Class 4 obesity	0.00827	0.00509	0.650	-0.0055	0.0221
	Class 3 obesity	Normal body fat	-0.00436	0.00381	1.000	-0.0147	0.0060
		Class 1 and 2 obesity	-.01474*	0.00543	0.049	-0.0294	0.0000
		Class 4 obesity	-0.00647	0.00326	0.303	-0.0153	0.0023
	Class 4 obesity	Normal body fat	0.00211	0.00332	1.000	-0.0069	0.0111
		Class 1 and 2 obesity	-0.00827	0.00509	0.650	-0.0221	0.0055
		Class 3 obesity	0.00647	0.00326	0.303	-0.0023	0.0153
TCD8+CD28+ lymphocytes (cel/μL)	Normal body fat	Class 1 and 2 obesity	-0.54982	0.32411	0.565	-1.4295	0.3299
		Class 3 obesity	-.98592*	0.23624	0.001	-1.6271	-0.3447
		Class 4 obesity	-.87382*	0.19942	0.000	-1.4151	-0.3326
	Class 1 and 2 obesity	Normal body fat	0.54982	0.32411	0.565	-0.3299	1.4295
		Class 3 obesity	-0.43610	0.32914	1.000	-1.3294	0.4572
		Class 4 obesity	-0.32400	0.30380	1.000	-1.1486	0.5006
	Class 3 obesity	Normal body fat	.98592*	0.23624	0.001	0.3447	1.6271
		Class 1 and 2 obesity	0.43610	0.32914	1.000	-0.4572	1.3294
		Class 4 obesity	0.11210	0.20749	1.000	-0.4511	0.6753
	Class 4 obesity	Normal body fat	.87382*	0.19942	0.000	0.3326	1.4151
		Class 1 and 2 obesity	0.32400	0.30380	1.000	-0.5006	1.1486
		Class 3 obesity	-0.11210	0.20749	1.000	-0.6753	0.4511
TCD8+CD28- lymphocytes (cel/μL)	Normal body fat	Class 1 and 2 obesity	0.02058	0.35791	1.000	-0.9513	0.9924
		Class 3 obesity	-0.62321	0.26087	0.118	-1.3316	0.0851
		Class 4 obesity	-0.49290	0.22132	0.175	-1.0938	0.1080
	Class 1 and 2 obesity	Normal body fat	-0.02058	0.35791	1.000	-0.9924	0.9513
		Class 3 obesity	-0.64379	0.36346	0.485	-1.6307	0.3431
		Class 4 obesity	-0.51348	0.33620	0.787	-1.4264	0.3994
	Class 3 obesity	Normal body fat	0.62321	0.26087	0.118	-0.0851	1.3316
		Class 1 and 2 obesity	0.64379	0.36346	0.485	-0.3431	1.6307
		Class 4 obesity	0.13031	0.23018	1.000	-0.4947	0.7553
	Class 4 obesity	Normal body fat	0.49290	0.22132	0.175	-0.1080	1.0938
		Class 1 and 2 obesity	0.51348	0.33620	0.787	-0.3994	1.4264
		Class 3 obesity	-0.13031	0.23018	1.000	-0.7553	0.4947
TCD4+CD45RA+ lymphocytes (cel/μL)	Normal body fat	Class 1 and 2 obesity	-0.53460	0.37261	0.933	-1.5444	0.4752
		Class 3 obesity	-0.62366	0.26347	0.123	-1.3377	0.0904
		Class 4 obesity	-.77599*	0.22714	0.006	-1.3916	-0.1604
	Class 1 and 2 obesity	Normal body fat	0.53460	0.37261	0.933	-0.4752	1.5444
		Class 3 obesity	-0.08906	0.37261	1.000	-1.0988	0.9207
		Class 4 obesity	-0.24139	0.34787	1.000	-1.1841	0.7013
	Class 3 obesity	Normal body fat	0.62366	0.26347	0.123	-0.0904	1.3377
		Class 1 and 2 obesity	0.08906	0.37261	1.000	-0.9207	1.0988

		Class 4 obesity	-0.15232	0.22714	1.000	-0.7679	0.4632
	Class 4 obesity	Normal body fat	.77599*	0.22714	0.006	0.1604	1.3916
		Class 1 and 2 obesity	0.24139	0.34787	1.000	-0.7013	1.1841
		Class 3 obesity	0.15232	0.22714	1.000	-0.4632	0.7679
TCD4+CD45RA+CD45RO+ lymphocytes (cel/µL)	Normal body fat	Class 1 and 2 obesity	-0.00238	0.01108	1.000	-0.0324	0.0277
		Class 3 obesity	.02623*	0.00784	0.008	0.0050	0.0475
		Class 4 obesity	.03050*	0.00676	0.000	0.0122	0.0488
	Class 1 and 2 obesity	Normal body fat	0.00238	0.01108	1.000	-0.0277	0.0324
		Class 3 obesity	0.02861	0.01108	0.071	-0.0014	0.0587
		Class 4 obesity	.03288*	0.01035	0.013	0.0048	0.0609
	Class 3 obesity	Normal body fat	-.02623*	0.00784	0.008	-0.0475	-0.0050
		Class 1 and 2 obesity	-0.02861	0.01108	0.071	-0.0587	0.0014
		Class 4 obesity	0.00427	0.00676	1.000	-0.0140	0.0226
	Class 4 obesity	Normal body fat	-.03050*	0.00676	0.000	-0.0488	-0.0122
		Class 1 and 2 obesity	-.03288*	0.01035	0.013	-0.0609	-0.0048
		Class 3 obesity	-0.00427	0.00676	1.000	-0.0226	0.0140
TCD4+CD45RO+ lymphocytes (cel/µL)	Normal body fat	Class 1 and 2 obesity	0.05692	0.27846	1.000	-0.6975	0.8113
		Class 3 obesity	-.78441*	0.19690	0.001	-1.3178	-0.2510
		Class 4 obesity	-.48322*	0.16902	0.033	-0.9411	-0.0253
	Class 1 and 2 obesity	Normal body fat	-0.05692	0.27846	1.000	-0.8113	0.6975
		Class 3 obesity	-.84132*	0.27846	0.021	-1.5957	-0.0870
		Class 4 obesity	-0.54014	0.25950	0.245	-1.2431	0.1629
	Class 3 obesity	Normal body fat	.78441*	0.19690	0.001	0.2510	1.3178
		Class 1 and 2 obesity	.84132*	0.27846	0.021	0.0870	1.5957
		Class 4 obesity	0.30119	0.16902	0.472	-0.1567	0.7591
	Class 4 obesity	Normal body fat	.48322*	0.16902	0.033	0.0253	0.9411
		Class 1 and 2 obesity	0.54014	0.25950	0.245	-0.1629	1.2431
		Class 3 obesity	-0.30119	0.16902	0.472	-0.7591	0.1567
TCD8+CD45RA+ lymphocytes (cel/µL)	Normal body fat	Class 1 and 2 obesity	0.13030	0.31536	1.000	-0.7253	0.9859
		Class 3 obesity	-0.34252	0.22299	0.773	-0.9475	0.2625
		Class 4 obesity	-0.31086	0.19500	0.692	-0.8399	0.2182
	Class 1 and 2 obesity	Normal body fat	-0.13030	0.31536	1.000	-0.9859	0.7253
		Class 3 obesity	-0.47283	0.31536	0.829	-1.3284	0.3828
		Class 4 obesity	-0.44116	0.29622	0.845	-1.2448	0.3625
	Class 3 obesity	Normal body fat	0.34252	0.22299	0.773	-0.2625	0.9475
		Class 1 and 2 obesity	0.47283	0.31536	0.829	-0.3828	1.3284
		Class 4 obesity	0.03167	0.19500	1.000	-0.4974	0.5607
	Class 4 obesity	Normal body fat	0.31086	0.19500	0.692	-0.2182	0.8399
		Class 1 and 2 obesity	0.44116	0.29622	0.845	-0.3625	1.2448
		Class 3 obesity	-0.03167	0.19500	1.000	-0.5607	0.4974
TCD8+CD45RA+CD45RO+ lymphocytes (cel/µL)	Normal body fat	Class 1 and 2 obesity	-0.00580	0.01132	1.000	-0.0365	0.0249

		Class 3 obesity	0.01350	0.00806	0.589	-0.0084	0.0354
		Class 4 obesity	0.01678	0.00705	0.119	-0.0023	0.0359
	Class 1 and 2 obesity	Normal body fat	0.00580	0.01132	1.000	-0.0249	0.0365
		Class 3 obesity	0.01930	0.01124	0.541	-0.0112	0.0498
		Class 4 obesity	0.02257	0.01053	0.213	-0.0060	0.0511
	Class 3 obesity	Normal body fat	-0.01350	0.00806	0.589	-0.0354	0.0084
		Class 1 and 2 obesity	-0.01930	0.01124	0.541	-0.0498	0.0112
		Class 4 obesity	0.00327	0.00691	1.000	-0.0155	0.0220
	Class 4 obesity	Normal body fat	-0.01678	0.00705	0.119	-0.0359	0.0023
		Class 1 and 2 obesity	-0.02257	0.01053	0.213	-0.0511	0.0060
		Class 3 obesity	-0.00327	0.00691	1.000	-0.0220	0.0155
TCD8+CD45RO+ lymphocytes (cel/µL)	Normal body fat	Class 1 and 2 obesity	-0.42756	0.27498	0.746	-1.1733	0.3182
		Class 3 obesity	-1.06312*	0.19444	0.000	-1.5905	-0.5358
		Class 4 obesity	-.93751*	0.16919	0.000	-1.3964	-0.4787
	Class 1 and 2 obesity	Normal body fat	0.42756	0.27498	0.746	-0.3182	1.1733
		Class 3 obesity	-0.63556	0.27498	0.142	-1.3813	0.1102
		Class 4 obesity	-0.50995	0.25775	0.310	-1.2090	0.1891
	Class 3 obesity	Normal body fat	1.06312*	0.19444	0.000	0.5358	1.5905
		Class 1 and 2 obesity	0.63556	0.27498	0.142	-0.1102	1.3813
		Class 4 obesity	0.12561	0.16919	1.000	-0.3332	0.5845
	Class 4 obesity	Normal body fat	.93751*	0.16919	0.000	0.4787	1.3964
		Class 1 and 2 obesity	0.50995	0.25775	0.310	-0.1891	1.2090
		Class 3 obesity	-0.12561	0.16919	1.000	-0.5845	0.3332

*. The difference in means is significant at the 0.05 level.

9- To determine relationships between lymphocyte subpopulations and metabolic variables, we utilized a Pearson correlation matrix and identified correlations greater than ± 0.6. We considered $p < 0.05$ to indicate a statistically significant difference. We used the Phyton Environment v3.6.7 statistical package (CreateSpace 2009, USA). (Table 17S)

Table 17.1S. Correlation between immunological variables and the metabolic and clinical variables of the entire study group

Var X	Var y	r value	p value
TBF (%)	BMI (kg/m2)	0.879970	8.31E-30
BMI (kg/m2)	TCD8+CD45RO+ lymphocytes (cel/µL)	0.686432	3.15E-08
WC (cm)	TCD8+CD45RO+ lymphocytes (cel/µL)	0.673646	5.28E-08
Weight (kg)	TCD8+CD45RO+ lymphocytes (cel/µL)	0.519938	1.26E-06
TBF (%)	TCD8+CD45RO+ lymphocytes (cel/µL)	0.50041	3.58E-06
BMI (kg/m2)	TCD4+CD62- lymphocytes (cel/µL)	0.526419	1.93E-07
CRP (mg/dL)	TCD4+ lymphocytes (cel/µL)	0.528084	2.44E-07
Weight (kg)	Total lymphocytes (cel/µL)	0.413708	7.52E-05
Weight (kg)	TCD4+ lymphocytes (cel/µL)	0.407462	9.87E-05
Weight (kg)	TCD4+CD62- lymphocytes (cel/µL)	0.446095	1.67E-05
Weight (kg)	TCD4+CD45RO+ lymphocytes (cel/µL)	0.407349	0.000177
Weight (kg)	TCD8+CD45RO+ lymphocytes (cel/µL)	0.419938	1.26E-06
WC (cm)	TCD8+CD45RO+ lymphocytes (%)	0.422778	1.3E-05
WC (cm)	Leukocytes (cel/µL)	0.400363	0.000147

WC (cm)	Total lymphocytes (cel/µL)	0.451846	1.26E-05
WC (cm)	TCD3+ lymphocytes (cel/µL)	0.444684	2.01E-05
WC (cm)	TCD4+CD62- lymphocytes (cel/µL)	0.481465	2.69E-06
TBF (%)	TCD8+ lymphocytes (%)	0.402567	6.81E-06
TBF (%)	TCD4+ lymphocytes (cel/µL)	0.443611	1.89E-05
TBF (%)	TCD4+CD62- lymphocytes (cel/µL)	0.457679	9.4E-06
TBF (%)	TCD8+CD45RO+ lymphocytes (cel/µL)	0.50041	3.58E-06
BMI (kg/m2)	Total lymphocytes (cel/µL)	0.418067	6.19E-05
BMI (kg/m2)	TCD3+ lymphocytes (cel/µL)	0.419928	6.31E-05
BMI (kg/m2)	TCD4+ lymphocytes (cel/µL)	0.495786	1.21E-06
BMI (kg/m2)	TCD4+CD62- lymphocytes (cel/µL)	0.526419	1.93E-07
BMI (kg/m2)	TCD4+CD45RA+CD45RO+ lymphocytes (cel/µL)	-0.43527	6.1E-05
BMI (kg/m2)	TCD3+ lymphocytes (cel/µL) (para raro cd8)	0.450166	1.54E-05

Table 17.2S. Correlation between immunological variables and the metabolic and clinical variables of the class 1 and 2 obesity

Var X	Var y	r value	p value
FFM (%)	TCD4+CD45RO+ lymphocytes (%)	0.769907	0.000192
FFM (%)	TCD4+CD45RA+ lymphocytes (%)	-0.70408	0.001109
Age (years)	TCD3 lymphocytes (%)	0.70156	0.014328
FFM (%)	TCD4+CD45RO+ lymphocytes (%)	0.568907	0.000192
FFM (%)	Monocytes (%)	0.551761	0.006344
LDL-c (mg/dL)	Age (years)	0.405323	0.044423
Age (years)	NK lymphocytes (%)	0.477629	0.028544
FFM (kg)	TCD4+CD45RO+ lymphocytes (%)	-0.47217	0.013092
Glucose (mg/dL)	TCD4+CD45RO+ lymphocytes (%)	-0.40020	0.034515
HDL-c (mg/dL)	TCD8+ lymphocytes (%)	0.423193	0.044213

Table 17.3S. Correlation between immunological variables and the metabolic and clinical variables of the 3 obesity

Var X	Var y	r value	p value
CRP (mg/dL)	Granulocytes (cel/µL)	0.762016	0.002708
CRP (mg/dL)	Leukocytes (cel/µL)	0.725194	0.000200
VF (cm^2)	Leukocytes (cel/µL)	0.727146	0.007743
VF (cm^2)	TCD8+CD45RA+CD45RO+ lymphocytes (cel/µL)	-0.61231	0.008895
FFM (%)	TCD4+CD45RA+ lymphocytes (%)	-0.42960	0.046004
FFM (%)	Leukocytes (cel/µL)	0.534213	0.012609
FFM (%)	Total lymphocytes (cel/µL)	0.456551	0.037486
FFM (%)	TCD3+ lymphocytes (cel/µL)	0.446847	0.042275
Total cholesterol (mg/dL)	TCD8+CD45RO+ lymphocytes (%)	0.450067	0.021051
Total cholesterol (mg/dL)	Age (years)	0.456780	0.009790
Total cholesterol (mg/dL)	TCD8+ lymphocytes (%)	0.402137	0.030576
Age (years)	TCD4+CD45RA+CD45RO+ lymphocytes (%)	0.426000	0.037928
Age (years)	TCD4+CD45RA+CD45RO+ lymphocytes (cel/µL)	-0.48861	0.039636
BMI (kg/m2)	NK lymphocytes (%)	0.437265	0.017693

BMI (kg/m2)	TCD4+CD45RA+CD45RO+ lymphocytes (%)	-0.49843	0.013175
Glucose (mg/dL)	TCD4+CD45RA+ lymphocytes (%)	0.452482	0.034478
Glucose (mg/dL)	Granulocytes (cel/µL)	-0.47543	0.029390
CRP (mg/dL)	TCD4+ lymphocytes (cel/µL)	0.470103	0.031519

Table 17.4S. Correlation between immunological variables and the metabolic and clinical variables of the 3 obesity			
**Var X**	**Var y**	**r value**	**p value**
TBF (%)	TCD4+CD45RO+ lymphocytes (%)	0.646581	0.002548
Age (years)	TCD8+CD28- lymphocytes (cel/µL)	-0.62224	0.001534
LDL-c (mg/dL)	Age (years)	0.461274	0.002108
Age (years)	TCD8+ lymphocytes (cel/µL)	0.407478	0.01111
Age (years)	TCD8+ lymphocytes (cel/µL)	0.413918	0.008807
CRP (mg/dL)	TCD3+ lymphocytes (cel/µL)	0.456071	0.003523
