# Supplementary figures and images for: The link between lymphocyte subpopulations in peripheral blood and metabolic variables in patients with severe obesity
Source: PeerJ. 2023 Jun 13;11:e15465. doi: 10.7717/peerj.15465 (PMC10274585; doi:10.7717/peerj.15465)

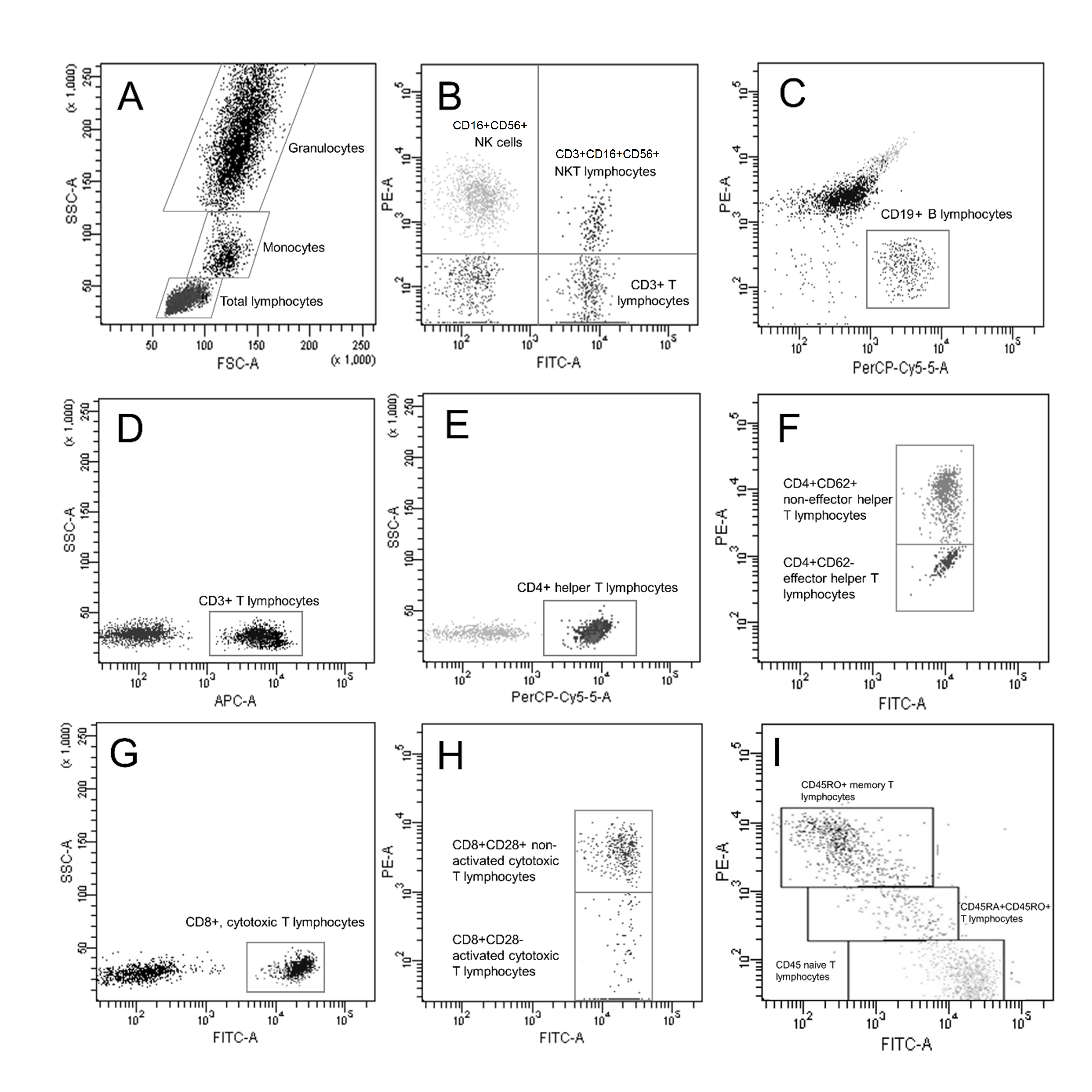

Supplement: Figure S1 — We utilized the following combinations of conjugated antibodies: (1SA) Control isotype with forward scatter (FSC) determines cell size and side scatter (SSC) determines complexity cell. We use FSC/SSC to identifies by morphology total lymphocytes, monocytes, and granulocytes. Lymphocytes are small and slightly granular cells, and they are represented at the bottom of the dot plot. They are followed by monocytes with a larger size and granularity and finally, granulocytes, which are the largest and most complex cells. (1SB & C) FITC-anti-CD3/PE-anti-(CD16+CD56)/PerCP-anti-CD19 (identifies T lymphocytes, natural killer [NK] cells, and B lymphocytes). (1S D, E & F) FITC-anti-CD4/PE-anti-CD62L/APC-anti-CD3 (identifies activated T lymphocytes with helper functions). (1SD, G & H) FITC-anti-CD8/PE-anti-CD28/APC-anti-CD3 (identifies activated T lymphocytes with cytotoxic functions). (1SD & I) FITC-anti-CD45RA/PE-anti- CD45RO/PerCP-anti-CD4/APC-anti-CD3 (identifies naïve helper T lymphocytes and memory T lymphocytes). (1S D & I) FITC-anti-CD45RA/PE-anti-CD45RO/PerCP-anti- CD8/APC-anti-CD3 (identifies cytotoxic naïve and memory T lymphocytes). [file peerj-11-15465-s003.png]
